# Effects of Small-Sided Soccer Games on Physical Fitness and Cardiometabolic Health Biomarkers in Untrained Children and Adolescents: A Systematic Review and Meta-Analysis

**DOI:** 10.3390/jcm13175221

**Published:** 2024-09-03

**Authors:** Nicolás Gómez-Álvarez, Giorjines Boppre, Felipe Hermosilla-Palma, Tomás Reyes-Amigo, José Oliveira, Hélder Fonseca

**Affiliations:** 1 Centre for Research, Education, Innovation and Intervention in Sport, Faculty of Sport, University of Porto, 4200-450 Porto, Portugal; 2 Nucleus of Research in Human Motricity Sciences, Universidad Adventista de Chile, Chillán 3780000, Chile; 3 Research Centre in Physical Activity, Health, and Leisure (CIAFEL), Faculty of Sport, University of Porto, 4200-450 Porto, Portugal; giorjines.boppre@gmail.com (G.B.); joliveira@fade.up.pt (J.O.); hfonseca@fade.up.pt (H.F.); 4 Laboratory for Integrative and Translational Research in Population Health (ITR), 4050-600 Porto, Portugal; 5 Pedagogía en Educación Física, Facultad de Educación, Universidad Autónoma de Chile, Talca 3460000, Chile; felipe.hermosilla@uautonoma.cl; 6 Physical Activity Sciences Observatory (OCAF), Department of Physical Activity Sciences, Universidad de Playa Ancha, Valparaíso 2360072, Chile; tomas.reyes@upla.cl

**Keywords:** cardiometabolic risk factors, sports, exercise, pediatric obesity, metabolic syndrome

## Abstract

**Objective:** This systematic review and meta-analysis aimed to determine the benefits of an exercise intervention based on small-sided soccer games (SSSGs) on health-related physical fitness and cardiometabolic health in previously untrained children and adolescents. **Methods:** A systematic search on PubMed/MEDLINE, Web of Science, Scopus, Cochrane, and EBSCO databases was performed. Randomized or non-randomized controlled trials conducted in previously untrained children or adolescents (age < 18 years) that assessed the effect of SSSG-based intervention on health-related physical fitness and cardiometabolic risk biomarkers were included. Primary outcomes were cardiorespiratory fitness and waist circumference. Evidence was synthesized as the mean difference or standardized mean difference using a random-effects meta-analysis. The quality of evidence was assessed using ROB2 and ROBINS-I tools. **Results:** Sixteen studies (n = 2872 participants) were included in this meta-analysis. SSSGs significantly improved cardiorespiratory fitness (SMD, 0.12 [0.01; 0.23]) and showed a non-significant trend in decreased waist circumference (−7.49 cm [−15.03; 0.06]). Additionally, SSSGs significantly decreased systolic (MD, −3.85 mmHg [−5.75; −1.94]) and diastolic blood pressure (MD, −1.26 mmHg [−2.44; −0.08]) and triglycerides (−30.34 mg·dL^−1^ [−45.99; −14.69]). No effects on body composition or other cardiometabolic risk biomarkers were observed. After a sensitivity analysis, waist circumference and muscle strength were also shown to improve significantly following SSSGs. Comparisons between SSSG and other types of exercise interventions showed no differences in improved physical fitness or cardiometabolic risk. **Conclusions:** SSSG-based interventions effectively improve cardiorespiratory fitness, blood pressure, triglycerides, muscle strength, and waist circumference. There is less evidence of the effects of SSSGs on other health markers. Particular attention should be given to improving SSSG protocol reporting in future studies.

## 1. Introduction

Obesity in children and adolescents is one of the most critical public health problems worldwide, and it is estimated that between 1990 and 2022, its prevalence has more than doubled in several countries [1]. The negative effect of excess weight is associated, among other things, with cardiometabolic complications [2,3,4,5,6,7,8]. It is estimated that the worldwide prevalence of metabolic syndrome in the pediatric population is approximately 3%, increasing to 11.9% in overweight and 29.2% in children with obesity [9], which implies a significant burden of cardiovascular risk early from infancy [10]. Evidence shows that the aggregation of cardiometabolic risk factors since childhood increases the burden of cardiovascular and metabolic disease in adulthood [2,3,11].

Physical exercise is an important part of the prevention and treatment of obesity and associated comorbidities [12,13]. Systematic reviews have suggested that physical exercise is associated with decreases in adiposity measures, reductions in blood pressure, and improvements in lipid profile and glucose metabolism [14]. However, high dropout rates and low adherence are frequent problems in exercise interventions [12]. Recently, sports-based strategies have been proposed to prevent and treat childhood obesity, with several studies highlighting the potential of soccer in this regard [15,16,17,18,19]. In particular, small-sided soccer games (SSSGs) have been proposed as an appropriate strategy for health promotion in children because they allow performing high-intensity exercise [17,18,19] while also being a social, enjoyable, and inexpensive activity [15,20], which might increase long-term adherence to exercise.

Training based on SSSG has been widely studied in athletes of all ages since it allows, through different game restrictions, the physiological demands of exercise to be regulated, potentially eliciting improvements in physical performance comparable to those of traditional training methods [20]. Studies have suggested that the intensity during SSSGs would be similar to that during high-intensity interval exercise but with a higher perceived enjoyment [21]. A systematic review by Zouhal et al. [22] on the acute and long-term effects of SSSGs on physical fitness and health outcomes concluded that SSSGs can elicit high cardiovascular and metabolic demands that, in the long term, could enhance physical fitness and health biomarkers. Recently, Clemente et al. [15] suggested that recreational soccer, in the form of SSSGs, could be an effective strategy to improve cardiorespiratory fitness, decrease blood pressure, and improve health indicators, but there are inconsistent results on the effects on body composition or other measures of cardiometabolic risk.

Furthermore, despite the evidence and growing interest in SSSGs as a strategy for enhancing cardiometabolic health, systematic reviews have not included meta-analysis assessing its potential effect on improving cardiometabolic health in children and adolescents. Therefore, the main objective of this study is to determine the type and magnitude of the benefits of SSSG exercise interventions on health-related physical fitness and cardiometabolic risk biomarkers in untrained children and adolescents. Secondary objectives are to (i) compare the effectiveness of SSSGs vs. other exercise interventions on the improvement of health-related physical fitness and cardiometabolic health and (ii) investigate the effect of age, obesity severity, and duration of the SSSG exercise intervention on potential SSSG-induced benefits.

## 2. Materials and Methods

The systematic review followed the recommendations and criteria established by the Preferred Reporting Items for Systematic Reviews and Meta-analyses (PRISMA) reporting guidelines [23]. The protocol was registered in March 2021 at the International Prospective Register of Systematic Reviews (PROSPERO) with the identifier code CRD42021233308.

### 2.1. Eligibility Criteria

The PICOS approach (Population, Intervention, Comparator, Outcomes, Study design) was used to define inclusion and exclusion criteria. Studies that meet the following criteria were included:

Participants: Studies carried out in children and adolescents (<18 years of age), healthy or associated with health conditions, were included. Studies with young soccer players, children, adolescents regularly engaged in competitive sports (participation in sports clubs that conduct systematic training sessions with the objective of improving sports performance), or adults (>18 years) were excluded.

Intervention: Studies with exercise interventions based on SSSGs as the central part of the intervention and that presented results on at least one parameter related to cardiometabolic health or health-related physical fitness were included. Studies with exercise interventions other than SSSGs or soccer-based interventions that were not organized as SSSGs were excluded.

Comparator: Studies comparing the effects of SSSG-based interventions with (i) a control group that continued their activities of daily living without an additional exercise intervention and (ii) an exercise intervention group undergoing exercise interventions other than SSSGs. Studies that did not include a control group were excluded.

Outcome: Studies reporting an assessment of the long-term (≥8-week) effects of an SSSG-based intervention on measures of health-related fitness (cardiorespiratory or muscular fitness), health-related biomarkers (fasting blood glucose [mg/dL]; triglycerides [mg/dL]; HDL cholesterol [mg/dL]; LDL cholesterol [mg/dL]; total cholesterol [mg/dL]; HOMA-IR; leptin [ng mL^−1^], IL-6 [pg mL^–1^]; adiponectin [µg mL^−1^]; C-reactive protein [mg mL^−1^]; tumor necrosis factor-α [pg mL^–1^]; resistin [ng mL^−1^]), blood pressure [mmHg], resting heart rate [bpm]) and anthropometry and body composition (waist circumference [cm]; BMI [kg/m^2^]; body mass [kg]; fat mass; lean mass) were included. Studies that did not present data on cardiometabolic risk or health-related physical fitness were excluded.

Study type: Randomized controlled trials (RCT) or non-randomized controlled trials (non-RCT) published in English, Portuguese, or Spanish. Experimental studies without a control group, non-experimental studies, literature reviews, grey literature, or studies on experimental animals were excluded.

### 2.2. Outcome and Prioritization

Cardiorespiratory fitness and waist circumference were defined as primary outcomes to represent changes in physical fitness and cardiometabolic risk factors, respectively.

Other measures related to physical fitness (muscular fitness) and other cardiometabolic risk factors were defined as secondary outcomes.

### 2.3. Literature Search Strategy

A literature search was initially conducted in May 2023 in five electronic databases, including PubMed/MEDLINE, Web of Science, Scopus, Cochrane, and EBSCO, for articles published in English, Spanish, or Portuguese from inception to May 2023. The search strategy combined relevant keywords related to population (e.g., Child OR Adolescent OR Childhood OR Children), type of intervention (e.g., Soccer OR Small-sided Games OR Football OR “SSSG”), and outcomes of interest (e.g., measures related to metabolic syndrome (MetS), health-related physical fitness or other biomarkers of cardiometabolic risk), combined using boolean operators such as “AND”, “OR” and “NOT” and truncated terms. Snowballing was also performed. All search strategies are detailed in the Supplemental Materials (Methods S1).

### 2.4. Study Selection

All references were exported to EndNote 20.1 (Thomson and Reuters, San Francisco, CA, USA), and duplicates were removed. Two authors independently completed the eligibility assessment, first by title and abstract analysis in EndNote and, afterward, by full-text assessment from the journal website in which each article was published. If multiple reports for the same study were identified during the full-text review, reports that did not present new relevant results were excluded, while the report containing all relevant results was included. In disagreements between reviewers, consensus was reached with the help of a third reviewer.

### 2.5. Data Extraction

A form adapted from “The Cochrane Consumers and Communication Review Group’s data extraction standardized” was used. When multiple overlapping reports from the same study were identified, the information from the one containing the most relevant information (1st criterion) or the first published report (2nd criterion) was included.

The following data items were extracted: authors, country, publication year, study design, sample size, participants’ characteristics (age, sex, maturity stage, body mass, nutritional status or comorbidities), SSSGs and comparator intervention characteristics (SSSG model, volume, intensity, frequency, intervention type, duration). When results were reported in multiple time points, only results at baseline and end of the intervention were used.

### 2.6. Assessment of Risk of Bias

Two reviewers independently examined the methodological quality of the included studies using the Cochrane Risk of Bias (ROB2) [24] and the Risk of Bias in Non-Randomized Studies of Interventions (ROBINS-I) [25] tools for randomized and non-randomized studies, respectively. Any disagreements were resolved by discussion with a third author. These results are presented by individual and global plots representation [26].

### 2.7. Statistical Analysis

A meta-analysis was performed using the meta [27] and dmetar [28] packages in R version 3.4.3 (R Core Team, Vienna, Austria). The pooled effect of the SSSG exercise interventions on the selected outcomes was examined using a random-effects meta-analysis (DerSimonian-Laird approach) [29]. Analyses were conducted when at least three studies investigating an outcome of interest were available. Whenever the number of studies reporting an outcome of interest was insufficient, only a qualitative analysis of the results was performed.

The mean difference of the change between pre and post-intervention and standard deviation (SD) of each variable of interest in the SSSG and comparator group were determined to calculate effect sizes. The transformation methods suggested in the Cochrane Handbook [30] were followed if these data were not reported. Effect sizes were expressed as the mean difference (MD) and 95% confidence interval. When the results were measured with different scales or with different units of measurement, they were expressed as the standardized mean difference (SMD) and 95% confidence interval. To correct for possible small sample bias when calculating the SMD, we calculated Hedges’ g. The effect sizes were considered large, medium, small, or trivial when the SMD was >0.8, 0.5 to 0.8, 0.2 to 0.5, or below 0.2, respectively.

The I2 statistic assessed heterogeneity, and the following cut-off values used for interpretation: <25, 25–50, and >50% were considered small, medium, and large heterogeneity, respectively [31]. For all outcomes, sensitivity analyses according to the leave-one-out method were performed to determine the influence of individual studies on the overall effect, using diagnostic plots proposed by Viechtbauer and Cheung [32]. Egger’s regression test was used to examine publication bias when 10 or more reports with the same outcome were available [33]. Whenever possible, subgroup analyses were performed for primary outcomes considering age (<12 years or >12 years), sex, nutritional status, duration of the intervention (<20 weeks or >20 weeks), and study design.

## 3. Results

### 3.1. Study Selection

**Figure 1:**
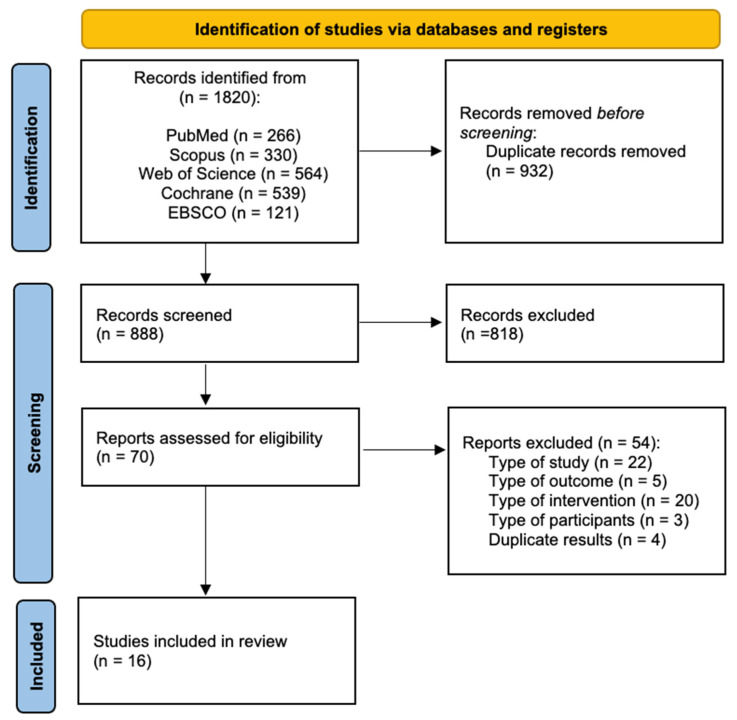
Flow chart for identification of reports for inclusion in the systematic review and meta-analysis.

A total of 1820 articles were initially retrieved from the literature search. After duplicate removal, 888 studies were screened by title and abstract. From these, 70 were selected and their full text analyzed, from which 16 reports met all eligibility criteria (Figure 1). The final pool of reports selected included five RCTs [34,35,36,37,38], seven cluster RCTs [39,40,41,42,43,44,45], and four non-RCTs [46,47,48,49].

### 3.2. Study Characteristics and Participants

The characteristics of the RCT and non-RCT are summarized in Table 1. Studies were performed in six different countries: five in Denmark [39,40,41,44,45], three in Portugal [47,48,49], two in Serbia [34,43], two in Brazil [37,38], and one in Germany [35], Tunisia [36], Faroes Islands [42], and Chile [46]. All studies implemented SSSGs as the main component of exercise intervention and compared it with a control group without any exercise intervention or with other types of exercise interventions. The total sample included 2872 participants (1944 in SSSG interventions, 134 in exercise programs other than SSSGs, and 794 in non-exercise control groups) between 8 and 17 years of age. Six studies included only boys [34,35,36,46,48,49], and ten studies included both boys and girls [37,38,39,40,41,42,43,44,45,46,47]. Five studies included only participants with overweight or obesity [34,35,46,47,48], two included only participants with obesity [37,49], and one included participants with obesity and metabolic syndrome [38], while in eight studies participants were included irrespective of nutritional status [39,40,41,42,43,44,45,46].

### 3.3. Small-Sided Soccer Games and Co-Intervention Characteristics

Fifteen of the included studies added an SSSG-based physical exercise intervention to the participants’ regular activities, and only in one study were regular physical education classes replaced by the SSSG exercise intervention [41]. SSSG programs lasted between 8 and 40 weeks, with eleven studies having 12 weeks or less [34,36,38,39,41,42,43,44,45,46,47] and five studies spanning 20 or more weeks [35,40,43,48,49]. The exercise frequency was between two and three times per week, and only one study included four sessions [47]. The duration of each session ranged from 45 to 60 min and contained the SSSG as the primary training load. Three studies defined the characteristics of the SSSG used, specifying the number of series, time of play, and rest [34,43,46]. In contrast, the remaining studies only described the total time in SSSGs [35,36,37,38,39,40,41,42,44,45,47,48,49]. The number of players was defined in twelve studies and varied between 2 and 7 per team. Six studies used only SSSG 3 vs. 3 [39,40,41,44,45,46], two studies employed 3 to 4 players per side [42,43], two studies 2 to 4 players per side [37,38], one study 4 to 7 players per side [36], and one study 5 to 7 players per side [34].

Four studies included another intervention group using other training modalities to compare SSSG effects versus other types of exercise. These additional training programs corresponded to multicomponent training [35,49], high-intensity interval training [34], or circuit training [40].

### 3.4. Outcome Measures

Fourteen studies assessing outcomes regarding health-related physical fitness and cardiometabolic risk biomarkers were included in the narrative synthesis and meta-analysis.

*Health-related physical fitness:* Seven studies estimated cardiorespiratory fitness (CRF) through either a yo-yo intermittent endurance test level 1 [34,36], yo-yo intermittent recovery children test [40,41,42,44,45], or yo-yo intermittent recovery test [43], in which the distance (meters) covered during the test corresponds to the CRF measure. Additionally, three studies used a metabolic cart to assess maximum/peak oxygen consumption during an incremental cycle ergometer [35,37] or treadmill test [49]. Muscle strength was determined by countermovement jump [34,35], vertical jump with a wall tape [43], or horizontal jump [36,41,42,44].

*Cardiometabolic risk biomarkers*: Anthropometric measures were evaluated in 11 studies. Body composition was assessed in ten studies: ten measured % fat mass [34,37,38,41,42,44,45,46,48,49], and seven measured lean mass [34,37,41,42,44,48,49] using bioelectrical impedance [34,41,42,44,45,46] or DXA [37,38,48,49]. Blood pressure was measured in 11 studies using either a manual sphygmomanometer [34] or an automatic upper-arm blood pressure monitor [36,37,38,39,40,41,42,45,47,49]. Biochemical markers of cardiovascular risk were assessed in three studies [37,38,49], with two studies including inflammation biomarkers [37,49].

**Table 1:** Characteristics of included studies on the systematic review and meta-analysis.

Author	Design	Participants			Intervention							Main Changes on Cardiometabolic Risk and Physical Fitness in SSSG (% Change)
		N	Age (Gender)	Nutrit. Status	w	Group (n)	d/w	Session (min)	Training Characteristics	Drop Out (%)	Adh (%)	
Carrasco et al., 2015 Chile [46]	CT	55	15.6 ± 0.7 (M)	OW-O	11	SSSG (NI)	2	75	SSSG F: 3 vs. 3; App: 50 m^2^; P-s: 20 × 15 m; S: 2; D-s: 15; W-d: 35 min; rest 5 min.	NI	90	%FM: ↓ 2.54 *; BMI: ↓ 0.61 *; VO_2max_.: ↑ 8.58 *
						CG (NI)			The normal level of physical activity	NI	95	
Cvetković et al., 2018 Serbia [34]	RCT	42	11 to 13 (M)	OW-O	12	SSSG (10)	3	60	SSSG F: 5–7 a side; App: 80 m^2^; S: 4; D-s: 8 min; W-d: 32 min; Rest: 2 min; W-i: 75.1 ± 2.3% HR_max_	28,57	>50	%FM: ↓ 7.67; LM: ↑ 2.61; BMI: ↓ 3.07; BM: ↓ 1.39; SBP: ↓ 2.89; DBP: ↓ 8.57; RHR: ↓ 10.20; CMJ: ↑ 17.02; YYIET1: ↑ 79.83 *
						HIIT (11)	3	60	HIIT; S: 3; Reps: 5–8–10; W-i: 100% MAS; rest: 3 min; W-i: 80.0 ± 3.0% HR_max_	21,43	>50	
						CG (14)			The normal level of physical activity	0		
Faude et al., 2010 Germany [35]	RCT	39	8 to 12 (M)	OW-O	24	SSSG (11)	3	60	SSSG (50%) + technique (20%) + fitness courses with the ball	57,89	>50	BM: ↑ 5.78 *; BMI: ↑ 1.86 *CMJ: ↑ 15.38 *; VO_2max_.: ↓ 6.72
						STD (11)	3	60	Aerobic endurance (40%) + coordination/flexibility (20%) + strength (15%) + speed (15%)	55	>50	
Hammami et al., 2017 Tunisia [36]	RCT	22	15.9 ± 0.6 (M)	N	8	SSSG (10)	2	30–45	SSSG F: 4–7 a side; P-s: 20 × 25 to 50 × 30 m.	9.09	NI	SBP: ↑ 1.3; DBP: ↓ 2.9; HRR: ↓ 6.5; HJ: ↑ 3.8; YYIRT1: ↑ 30.92 *
						CG (10)			The normal level of physical activity	9.09	NI	
Hansen et al., 2013 Portugal [47]	CT	31	8 to 12 (MF)	OW-O	12	SSSG (20)	4	45–90	SSSG + technical exercise; W-i: >90% HRmax; W-d: 40–60 min	0	NI	BM: ↑ 0.39; BMI: ↓ −0.87SBP: ↓ 1.75 *; DBP: ↑ 1.61; RHR: ↓ 4.48
						CG (11)			Normal level of physical activity	0	NI	
Krustrup et al., 2014 Denmark [39]	RCT	97	9 to 10 (MF)	N-OW	10	SSSG (46)	3	40	SSSG F; 3 vs. 3; W-d: 30 min; W-i: 71 ± 28% HRmax, time >80% HR_max_ = 24 ± 13%.	0	77 ± 18	BM: ↑ 1.56; BMI: ↓ −0.59SBP: ↓ 0.89; DBP: ↓ 1.34; RHR: ↓ −0.71
						CG (51)			Normal level of physical activity	0		
Larsen et al., 2018 Denmark [40]	cRCT	291	8 to 10 (MF)	N-OW	40	SSSG (93)	3	40	SSSG F: 3 vs. 3; P-s: 20 × 13m	NI	NI	BM: ↑ 8.26; SBP: ↓ 1.34; DBP: ↓ 3.13; RHR: ↓ 2.36; MAP: ↓ 2.38; YYIR1C: ↑ 19.76
						CST (83)	3	40	Circuit training; S: 6–10; D-s: 30 s; rest: 45 s.	NI	NI	
						CG (115)	3	40	Normal level of physical activity	NI	NI	
Ørntoft et al., 2016 Denmark [41]	cRCT	546	10 to 12 (MF)	N-OW	11	SSSG (386)	2	45	SSSG + football skill; SSG F: 3 vs. 3.	3.99	NI	BM: ↑ 2.18 *; BMI: ↓ 0.11; LM: ↑ 4.17 *; %FM: ↓ 3.72 *; SBP: ↓ 2.75 * DBP: ↓ 3.43 *; MAP: ↓ 2.53; RHR: ↓ 1.39YYIR1C: ↑ 5.16 *; HJ ↓ 0.85
						CG (140)			Normal level of physical activity	2.77	NI	
Seabra et al., 2014 Portugal [48]	CT	20	8 to 12 (M)	OW-O	20	SSSG (12)	2	60–90	SSSG + technical exercise: W-d: 40–60 min; W-i: >80% HR_max_	0	>85	BM: ↑ 5.37; BMI: ↑ 1.31; %FM: ↓ 2.39; LM: ↑ 5.0
						CG (8)			Normal level of physical activity	0	>85	
Seabra et al., 2016 Portugal [49]	CT	90	8 to 12 (M)	O	24	SSSG (29)	3	60–90	SSSG + technical exercise: W-d: 40–60 min; W-i: 78% HR_max_	3.33	>85	BM: ↓ 0.76; BMI: ↓ 0.84; WC: ↓ 5.02 *; %FM: ↓ 6.41 *; ↑ LM: 8.38 *; FBG: ↑ 3.47; HDL: ↑ 7.65 *; LDL: ↓ 12.85 *; TG: ↓ 22.57 *; TC: ↓ 6.99 *; SBP: ↑ 0.36; DBP: ↓ 6.77; VO_2max_.: ↑ 12.75 *
						AG (29)	3	60–90	Multicomponent training: aerobic endurance, coordination, balance, flexibility, and strength; W-d: 40–60 min; W-i: 75% HR_max_.	3.33	>85	
						CG (30)			Normal level of physical activity	0		
Skoradal et al., 2018 Faroe Islands [42]	cRCT	392	10 to 12 (MF)	N-OW	11	SSSG (229)	2	45	SSSG F: 3 vs. 3–4 vs. 4.	NI	NI	BM: ↑ 4.52 *; BMI: ↑ 2.07 *; %FM: ↓ 2.60 *; LM: ↑ 5.02 *; SBP: ↓ 3.06 *; DBP: ↑ 1.59 *; RHR: ↓ 1.32; YYIR1C: ↑ 18 *; HJ: ↑ 4.59.
						CG (100)			Normal level of physical activity	NI	NI	
Trajković et al., 2020 Serbia [43]	RCT	152	14 to 17 (MF)	N-OW	32	SSSG (54)	2	45	SSSG F: 3 vs. 3, 4 vs. 4; App: 40–70 m^2^; S: 4; D-s: 5 min; W-d: 32 min; Rest: 3 min; W-i: 85–99% HR_peak_.	20	>85	BM: ↓ 1.81; BMI: ↓ 3.81; YYIRT1: ↑ 2.22 *; VJ: ↑ 3.39 *.
						CG (51)			Normal level of physical activity	30	>85	
Vasconcellos et al., 2016 Brazil [37]	RCT	42	12 to 17 (MF)	O	12	SSSG (10)	3	60	SSSG F: 2–4 a side; W-d: 40 min; W-i: 84.5 ± 4.1% HRmax.	37.5	NI	BM: ↓ 5.35 *; BMI: ↓ 2.25 *; WC: ↓ 8.31 *; %FM: ↓ 5.35 *; LM: ↑ 4.16; FBG: ↓ 1.08; HDL: ↑ 32.74 *; LDL: ↓ 0.20; TG: ↓ 17.31 *; TC: ↓ 9.74 *; SBP: ↓ 3.91 *; DBP: ↓ 2.47; VO_2_ peak: ↑ 31.35 *.
						CG (10)			Normal level of physical activity	37.5	NI	
Vasconcellos et al., 2020 Brazil [38]	RCT	13	13 to 17 (MF)	O/MetS	12	SSSG (6)	3	60	SSSG F: 2–4 a side; W-d: 40 min	0	100	BM: ↓ 5.05; BMI: ↓ 0.33; WC: ↓ 11.79; %FM: ↓ 8.04; FBG: ↓ 18.51; HDL: ↑ 49.68 *; TG: ↓ 20.06 *; SBP: ↓ 5.19; DBP: ↓ 3.45
						CG (7)			Normal level of physical activity	0	100	
Ryom et al., 2022 Denmark [45]	cRCT	1122	11 to 12 (MF)	N-OW	11	SSSG-PA (644)	2	45	SSSG + football skill; SSG F: 3 vs. 3.	NI	NI	YYIR1C: ↑ 16.09; BM: ↑ 1.91; BMI: ↓ −0.65; %FM: ↓ −3.07; DBP: ↓ −2.31; SBP:↑ 0.36; RHR: ↓ −0.75
						SSSG-NPA(300)	2	45	SSSG + football skill; SSG F: 3 vs. 3.	NI	NI	YYIR1C: ↑ 23.36; BM: ↑ 2.19; BMI: ↓ −0.15; %FM: ↓ −2.49; DBP: ↓ −3.05; SBP: ↑ 0.87; RHR: ↓ −2.03
						CG-PA(122)			Normal level of physical activity	NI	NI	
						CG-NPA(56)			Normal level of physical activity	NI	NI	
Larsen et al., 2023 Denmark [44]	cRCT	127	10 to 12 (MF)	N-OW	11	SSSG (61)	2	45	SSSG + football skill; SSG F: 3 vs. 3.	NI	NI	LM: ↑ 3.96; %FM: ↓ −1.72; HJ: ↑ 2.39
						CG (47)			Normal level of physical activity	NI	NI	

Abbreviation: RCT, Randomized control trial; cRCT, Cluster randomized control trial; CT, non-randomized control trial; M, male; F, female; N, normal weight; OW, overweight; O, obesity; SSSG, small-sided soccer game; CG, control group; HIIT, high-intensity interval training; AG, Activity group; w, weeks; d/w, days per weeks; SSG F, small-sided games format; App, area per player; P-s, Pitch size; S, sets; D-s, sets duration; W-d, work duration, W-i, Work intensity, HRmax, Maximal heart rate; HR_peak_, Peak heart rate; NI, uninformed; Adh, adherence; BM, Body mass; BMI. Body mass index; WC, Waist circumference; %FM, % Fat mass; LM, Lean mass; SBP, Systolic blood pressure; DBP, Diastolic blood pressure; FBG, Fasting blood glucose; HDL, high-density lipoprotein; LDL, low-density lipoprotein; TG, triglycerides; TC, Total Cholesterol; CMJ, countermovement jump; YYIET1, Yo-Yo intermittent endurance test level 1; HJ, horizontal jump; YYIRT1, Yo-Yo intermittent recovery test level 1; YYIR1C, Yo-Yo intermittent recovery test level 1 children; VO_2max_, maximal oxygen consumption; VJ, vertical jump. ↓ decrease; ↑ increase; * Significant differences pre-post intervention.

### 3.5. Risk of Bias within Studies

**Figure 2:**
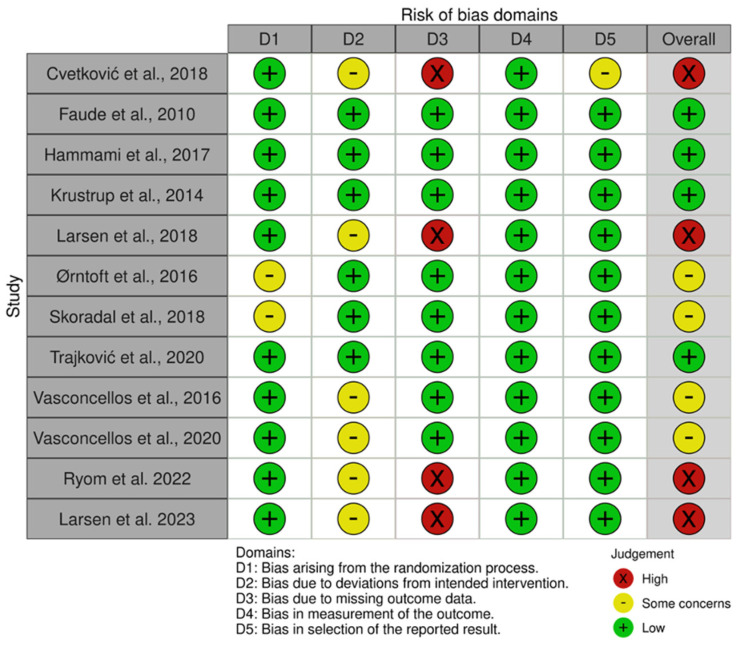
Assessment of risk of bias of randomized trials with RoB2.

**Figure 3:**
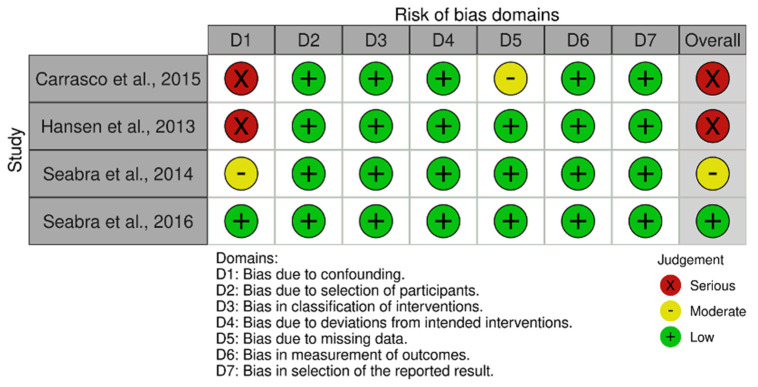
Assessment of risk of bias of non-randomized trials with ROBINS-I.

The summary of the risk of bias assessment for randomized and non-randomized studies is presented in Figure 2 and Figure 3. Analysis with ROB2 showed that two studies had a high risk of bias [34,40], and six studies had some concerns [37,38,41,42,44,45]. Bias assessment in non-randomized trials with the ROBINS-I tool found two high-risk studies [46,47], one moderate-risk study [48], and one low-risk study [49]. The plot with the overall results for RoB2 and ROBINS-I can be seen in the Supplementary Material (Results S1).

### 3.6. Summary of Results

**Figure 4:**
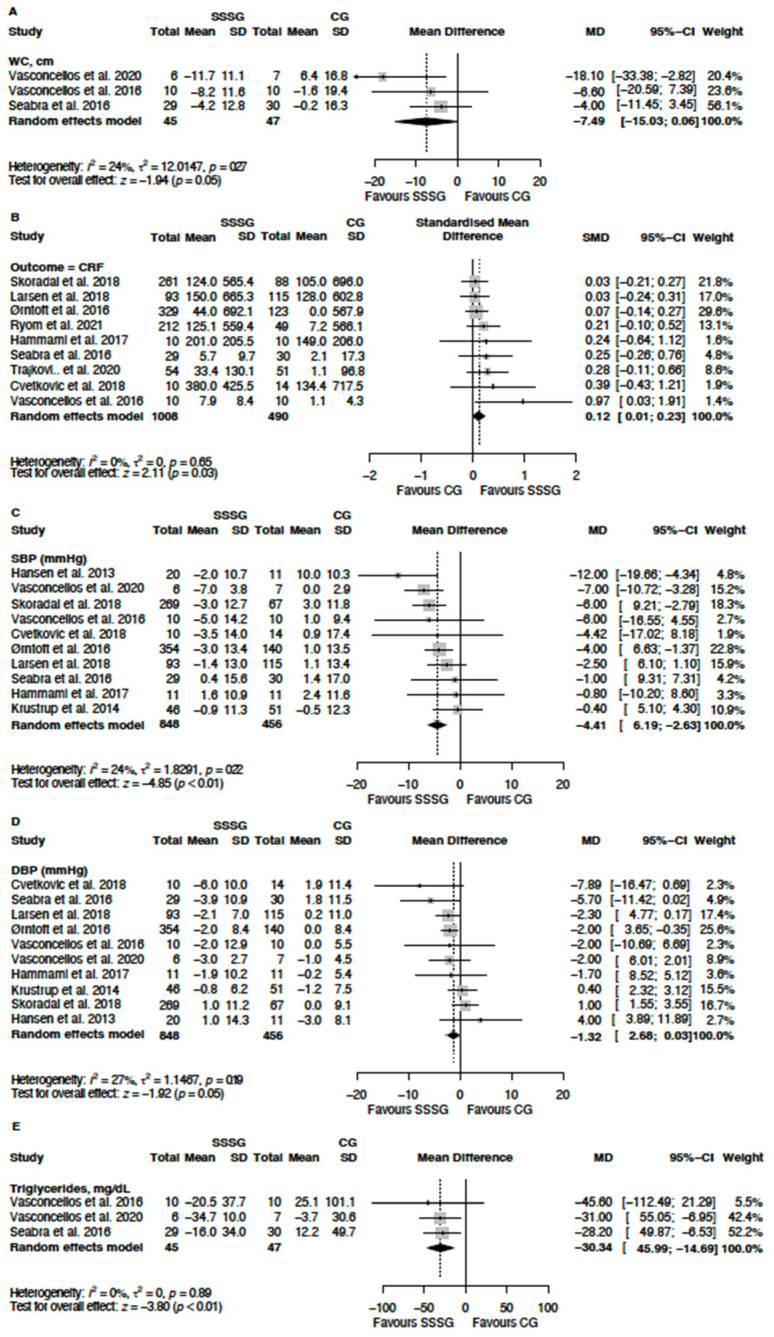
Forest plot of the mean difference between SSSGs and control groups for primary outcomes (**A**) waist circumference, (**B**) Cardiorespiratory fitness; and secondary outcomes (**C**) systolic blood pressure, (**D**) diastolic blood pressure and (**E**) blood triglycerides.

The summary of the meta-analysis for health-related physical fitness, anthropometry, body composition, and cardiometabolic variables for the comparisons between SSSGs and non-exercised controls and for the comparison between SSSGs and other interventions is presented in Table 2. Forest plots with the outcomes in which SSSG interventions presented significant differences relative to the control group are present in Figure 4.

*Primary outcomes:* The results of the meta-analysis showed that SSSGs have a significant effect on cardiorespiratory fitness improvement (SMD = 0.12; 95% CI = 0.01 to 0.23; I^2^ = 0%) and a non-significant trend towards a reduction in waist circumference (MD = −7.49 cm; 95% CI = −15.03 to 0.06; I^2^ = 24%). 

*Secondary outcomes:* Significant effects were found for systolic blood pressure (MD = −3.85 mmHg; 95% CI = −5.75 to −1.94; I^2^ = 38.8%), diastolic blood pressure (MD = −1.26 mmHg; 95% CI = −2.44 to −0.08; I^2^ = 38.8%) and blood triglycerides (MD = −30.34 mg·dL^−1^; 95% CI = −45.99 to −14.69; I^2^ = 0%) reduction (Table 2). The heterogeneity analysis revealed a moderate heterogeneity in muscle strength (I^2^ = 28%), systolic blood pressure (I^2^ = 38.8%) and resting heart rate (I^2^ = 25.5%), and high heterogeneity in fasting blood glucose (I^2^ = 45%). Forest plots for all analyses can be found in the Supplemental Materials.

Comparisons between SSSGs and other types of physical exercise interventions were also performed to determine their effects on cardiorespiratory fitness, body mass index, body mass, and systolic and diastolic blood pressure. Results showed no significant differences for any selected outcomes when SSSG interventions were compared with other exercise interventions (Supplemental Materials).

Table 3 summarizes the qualitative analysis of the physical fitness, cardiometabolic, and inflammatory outcome variables that were not included in the meta-analysis because of the need for a minimum number of reported outcomes. Only one study compared changes in muscle strength between SSSGs and other interventions, and no differences were identified [34]. Regarding anthropometric variables and body composition, when the effect of SSSGs was compared with that of other interventions, no differences in waist circumference [46], fat mass percentage [31,46], or lean mass [34,49] were identified.

Results for other biochemical markers showed that, in two studies, SSSGs significantly reduced total cholesterol [37,49]. The reduction was significantly higher than that of non-exercise controls but similar to other exercise interventions. The changes in LDL cholesterol, HOMA-IR, and fasting insulin showed inconsistencies between studies, and only HOMA-IR showed significant changes to non-exercised controls in one study [49]. There were also no differences in inflammation markers following SSSG interventions compared with non-exercised controls [37,49] or other exercise interventions [49].

**Table 3:** Qualitative synthesis of the effects of SSSGs on physical fitness, cardiometabolic risk, and inflammation markers.

Outcome	Variables	*k*	Individual Significant Findings	
			SSSG vs. CG	SSSG vs. Other Interventions
Physical fitness	Muscle strength	1	-	↔ Cvetkovic et al. [34]
Anthropometric and body composition	Waist circumference	1	-	↔ Seabra et al. [49] ^a^
	Body fat (%)	2	-	↔ Seabra et al. [49] ^a^ ↔ Cvetkovic et al. [34]
	Lean mass (kg)	2	-	↔ Seabra et al. [49] ^a^ ↔ Cvetkovic et al. [34]
Cardiometabolic and inflammatory Variables	Total cholesterol	2	↓ Seabra et al. [49] ^a^ ↓Vasconcellos et al. [37] ^a^	↔ Seabra et al. [49] ^a^
	LDL-C	2	↔ Seabra et al. [49] ^a^ ↔ Vasconcellos et al. [37]	↔ Seabra et al. [49] ^a^
	HDL-C	1	-	↔ Seabra et al. [49]
	TG	1	-	↔ Seabra et al. [49] ^a^
	HOMA-IR	2	↓ Vasconcellos et al. [37] ^a^ ↔ Seabra et al. [49]	↔ Seabra et al. [49]
	Fasting insulin	2	↔ Seabra et al. [49] ↔ Vasconcellos et al. [37]	↔ Seabra et al. [49]
	CRP	2	↔ Seabra et al. [49] ^a^ ↔ Vasconcellos et al. [37] ^a^	↔ Seabra et al. [49] ^a^
	Leptin	2	↔ Seabra et al. [49] ^a^ ↔ Vasconcellos et al. [37]	↔ Seabra et al. [49] ^a^
	Adiponectin	2	↔ Seabra et al. [49] ^a^ ↔ Vasconcellos et al. [37]	↔ Seabra et al. [49] ^a^
	IL-6	1	↔ Vasconcellos et al. [37]	
	Resistin	2	↔ Seabra et al. [49] ^a^ ↔ Vasconcellos et al. [37]	↔ Seabra et al. [49] ^a^
	TNF-α	1	↔ Vasconcellos et al. [37] ^a^	

Abbreviation: LDL-C, Low-density lipoprotein cholesterol; HDL-C, high-density lipoprotein cholesterol; TG, triglycerides; CRP, C-reactive protein; IL-6, Interleukin-6; TNF-α, Tumoral necrosis factor-α. ↔ no significant differences compared with a control group; ↓ significant decrease compared with the control group; ^a^ = significant pre-post-intervention differences in SSSG.

### 3.7. Subgroup Analyses

Subgroup analyses were performed to compare the effect of SSSGs versus non-exercised controls regarding systolic and diastolic blood pressure, cardiorespiratory fitness, and muscle strength, according to the following categories: age (≥12 or <12 years), sex (male only or male and female included), nutritional status (overweight/obesity, normal weight or overall irrespective of nutritional status), SSSG intervention duration (<20 or ≥20 weeks), and type of study design (RCT, cRCT, or non-RCT) (Supplemental Materials). Results showed no significant differences between the analyzed sub-groups (Supplementary, Results S4).

### 3.8. Publication Bias and Sensitivity Analysis

The Egger linear regression test and the funnel plot analysis showed no significant publication bias for outcome variables.

The detailed results of the leave-one-out analysis are presented in the Supplemental Materials. After the sensitivity analysis, the variables muscle strength (omitting Ørntoft et al. [41]; SMD = 0.26; 95% CI = 0.09 to 0.44; I^2^ = 0%), and waist circumference (omitting Seabra et al. [49]; MD = −11.92 cm (95% CI = −23.16 to −0.68; I^2^ = 16%) showed significant differences following an SSSG intervention compared with a non-exercised control group. The heterogeneity also decreased following the leave-one-out sensitivity analysis, as expected.

## 4. Discussion

The main objective of this systematic review and meta-analysis was to determine the benefits of SSSG-based exercise interventions on physical fitness and cardiometabolic risk biomarkers in untrained children and adolescents. The main findings were that SSSGs improved cardiometabolic health by increasing cardiorespiratory fitness and decreasing systolic and diastolic blood pressure and triglycerides compared with no exercise intervention. A non-significant trend of SSSGs toward a reduction in waist circumference was also identified. High heterogeneity was found between studies for some of the outcomes, and after sensitivity analysis, significant effects of SSSGs on waist circumference and muscle strength were also identified. In contrast, our results showed no significant differences between the effects of SSSG-based interventions and other exercise interventions on improving physical fitness and cardiometabolic health.

Previous systematic reviews have suggested that recreational soccer improves physical fitness and cardiometabolic health [15,16,19,22,50]; however, these reviews have not focused on SSSG-based training [16,19,22] or did not incorporate studies in children or adolescents [15,50]. Therefore, this is the first systematic review and meta-analysis to include a quantitative analysis of the effects of SSSG on physical fitness and cardiometabolic health.

The benefits of exercise on different cardiometabolic risk biomarkers in children and adolescents have been widely explored [12,13,51,52]. For example, a significant decrease in waist circumference of ~3 cm has been reported after an exercise intervention [12,13]. Our meta-analysis showed only a trend toward reducing waist circumference compared with a non-exercised control group. However, despite its clinical relevance and simplicity, only three studies included waist circumference measurements in their results [37,38,49], probably because these were the only studies that included children or adolescents with obesity. Indeed, the study included in our systematic review, which included only adolescents with obesity and metabolic syndrome, reported the most significant magnitude of change after the intervention [38].

Similarly, only three studies included biochemical measures of cardiometabolic risk [37,38,49]. Our meta-analysis showed a significant reduction in blood triglycerides, and two studies included in the qualitative synthesis reported significant decreases in total cholesterol compared with the non-exercise control group [37,49]. These results partially agree with previous reviews reporting a beneficial effect of aerobic exercise on blood triglycerides and other lipid profile measures in children with obesity. In contrast, interventions combining aerobic and resistance training have reported effects only on triglycerides [12,13]. Although it is recognized that the interaction between exercise and blood lipids is complex owing to several interconnected variables, it has been suggested that exercise characteristics such as intensity or volume could substantially mediate its effects on blood lipids [51]. More information is needed on the SSSG model and the type of workload used during training, which hinders a more thorough analysis of the load variables.

Blood pressure was one of the most reported measures in our review, with results consistent with those reported in reviews that analyzed the effects of physical exercise on blood pressure [12,13]. This suggests that SSSGs cause a significant decrease in blood pressure, with a greater magnitude in those who are overweight or obese. This is not unusual, as the potential of physical exercise to normalize blood pressure in high-risk populations is well-known [53]. Reviews focusing on children and adolescents with obesity have shown conclusive evidence of the benefits of physical exercise in reducing blood pressure in this population [12,13]. Still, no significant effects have been reported in normal-weight children [51].

Regarding physical fitness, the results of our meta-analysis revealed a trivial but significant effect on cardiorespiratory fitness improvement. Furthermore, after sensitivity analysis, we found, after omitting the study by Ørntoft et al. [41], a small but significant effect on muscle fitness.

Previous meta-analyses examining the effect of exercise on cardiorespiratory fitness have reported moderate [52,54,55] to large effects [50,56] but with high levels of heterogeneity. The studies selected for our systematic review displayed low heterogeneity, but individual effects ranged from trivial [41,42,44] to moderate [18,34,43,45,49] and large [37]. Previous systematic reviews identified that the causes of heterogeneity could be associated with methodological aspects (e.g., type of study design), type of exercise program (e.g., training modality, duration, intensity), and participant characteristics (e.g., age, sex, physical fitness at baseline, and nutritional status) [51,54,55]. Nevertheless, several subgroup analyses performed in our study showed that the response to SSSGs was similar irrespective of study characteristics, namely study design, age, sex, and participants nutritional, even though the magnitude of the response tended to be higher in adolescents (>12 years), studies that only included men, and studies with participants with overweight.

Regarding muscle fitness, we found an effect similar to that reported in previous reviews analyzing the impact of physical exercise [57]. However, this was identified only after omitting the study of Ørntoft et al. [41]. The influence of this study [41] was related to its weight within the meta-analysis and for being the study that reported the smaller magnitude of effect. Interestingly, Skoradal et al. [42] found opposite results in muscle fitness, using participants of the same age, the same experimental design, and the intervention program “FIFA 11 for Health”. However, the baseline characteristics of participants in Skoradal et al. [42] had, on average, lower fitness performance at the beginning of the study (Yo-Yo intermittent recovery level 1 children’s running test; horizontal jump performance), which could explain the higher response to training.

On the other hand, SSSGs did not show significant effects on some measures of metabolic risk, such as BMI, body composition (fat mass and lean mass), blood lipids (LDL cholesterol and HDL cholesterol), fasting blood glucose, and markers of systemic inflammation. In this regard, the characteristics of the participants included in some studies or the training protocols could limit the effect of measures associated with cardiometabolic risk. In particular, including participants with normal metabolic risk parameters [51,52] or in stages of growth and biological maturation [58,59,60] could misinterpret the differences observed between SSSG groups and controls. Of the studies in our systematic review, eight included apparently healthy participants (36–43); thus, identifying significant changes in body composition or biochemical measures of cardiometabolic risk after an exercise intervention is less likely. Furthermore, the adaptive potential of training during puberty is controversial [58,59]. Changes in body composition, increases in fitness, and decreases in insulin sensitivity may vary depending on the pubertal stage and participants’ sex. Indeed, systematic reviews of the effects of physical exercise in children or adolescents have shown contradictory effects associated with sex and age on cardiorespiratory fitness [51,54].

Differences in SSSG characteristics could also be related to suboptimal intensities to achieve higher fitness and cardiometabolic health benefits [51,61]. Some studies included in this review planned intensities above 80% of the maximum heart rate [43,47,48]. However, studies that measured, in fact, the intervention intensity reported exercise intensities between 71% and 78% of the maximum heart rate [37,39,49]. These possible differences between predicted and actual exercise intensity highlight the importance of improving the prescription and reporting characterization of SSSG-based training programs [62,63]. In fact, in this systematic review, we identified different models of SSSGs, suggesting a lack of clarity regarding prescription variables (e.g., court size, number of players, type of regimen, or implementation of rules), many of which are determinants to stimulate the achievement of vigorous-intensity zones which have been associated with more benefits on cardiometabolic health [61].

One of the secondary objectives of this review was to compare the effects of SSSGs with other types of physical exercise interventions. We compared SSSGs with other interventions, such as multicomponent training [35,49], high-intensity interval training [34], and circuit training [40]. Our results suggest that SSSGs induce similar physical fitness and cardiometabolic risk improvements compared with other types of exercise.

### Limitations and Practical Implications

Some limitations should be considered when interpreting the results of this systematic review and meta-analysis. The search strategy was considered an automated search in the main scientific databases available; however, snowballing was not performed. Results were limited to studies using a per-protocol approach. Lack of results from intention-to-treat approaches could reduce the finding’s external validity and applicability to real-world contexts, especially considering that SSSGs are proposed as a strategy to promote participation and adherence to exercise. Results of our meta-analysis on waist circumference and cardiometabolic risk biomarkers are also limited by the low availability of studies including these outcomes.

Physical activity and health professionals can find in SSSGs a versatile, low-cost strategy with a high social and recreational component that can be applied in programs to enhance the cardiometabolic health of children and adolescents. In addition, it could be an alternative to traditional exercise programs, with the possibility of being integrated into school physical education classes, extracurricular sports, and specific programs for managing obesity and cardiometabolic risk factors.

## 5. Conclusions

Our results suggest that SSSG-based training effectively improves cardiorespiratory fitness and has a possible beneficial effect on waist circumference reduction. Additionally, SSSGs can reduce blood pressure and triglyceride levels. However, other anthropometric measures, body composition, and cardiometabolic risk biomarkers were not shown to be significantly improved after SSSG interventions.

SSSGs could be an alternative to traditional exercise strategies since they were not shown to be inferior to other types of exercise interventions included in this study. Future studies incorporating SSSG programs should describe the program characteristics and implementation more thoroughly [62], allowing better analysis of the relationship between training loads and health outcomes achieved. Additionally, the risk of bias identified in these studies suggests the need for more randomized controlled trials with an improved experimental design.

## Figures and Tables

**Table 2:** Estimated effects of small-sided soccer games (SSSG) in opposition to the comparator groups in terms of physical fitness, anthropometry, body composition, and cardiometabolic variables.

		SSSG vs. CG				SSSG vs. Other Interventions				
	K (n)	MD/SMD [95% CI]	*p*-Value	I^2^%	Egger Test *p*-Value	K (n)	MD/SMD [95% CI]	*p*-Value	I^2^%	Egger Test *p*-Value
**PRIMARY OUTCOMES**										
WC, cm	3 (45/47)	−7.49 ^a^ [−15.03; 0.06]	0.05	24	-	-	-	-	-	-
Cardiorespiratory fitness	9 (108/490)	0.12 ^b^ [0.01; 0.23]	0.03	0	-	4 (143/134)	0.04 [−0.20; 0.27]	0.75	0	-
**SECONDARY OUTCOMES**										
***Health-related Physical fitness***										
Muscle strength	6 (725/333)	0.17 ^b^ [−0.01; 0.34]	0.06	28	-	-	-	-	-	-
**Cardiometabolic risk biomarkers**										
FBG, mg dL^−1^	3 (45/47)	−1.76 ^a^ [−8.73; 5.20]	0.62	62	-	-	-	-	-	-
TG, mg dL^−1^	3 (45/47)	−30.34 ^a^ [−45.99; −14.69]	<0.01	0	-	-	-	-	-	-
HDL, mg dL^−1^	3 (45/47)	4.42 ^a^ [−2.18; 11.03]	0.19	0	-	-	-	-	-	-
SBP, mmHg	11 (1073/506)	−3.85 ^a^ [−5.75; −1.94]	<0.001	38.8	0.98	3 (132/123)	−3.35 [−6.81; 0.10]	0.06	0	-
DBP, mmHg	11 (1073/506)	−1.26 ^a^ [−2.44; −0.08]	0.04	19.6	0.72	3 (132/123)	0.91 [−1.15; 2.97]	0.39	0	-
BMI, kg/m^2^	11 (1037/456)	−0.16 ^a^ [−0.63; 0.31]	0.50	0	0.04	3 (50/51)	−0.23 [−2.51;2,01] ^a^	0.84	0	-
Body mass, kg	12 (1130/571)	−0.29 ^a^ [−1.53; 0.95]	0.65	0	0.06	4 (143/134)	−0.59 [−3.31; 2.12] ^a^	0.67	0	-
Fat Mass, %	10 (728/318)	−0.10 ^b^ [−0.22; 0.01]	0.08	0	-	-	-	-	-	-
Lean Mass, kg	7 (757/333)	0.03 ^b^ [-0.10; 0.16]	0.62	0	-	-	-	-	-	-
RHR, bpm	8 (1028/459)	−0.84 ^a^ [−2.81; 1.13]	0.41	25.5	-	-	-	-	-	-

Abbreviation: BMI, body mass index; WC, waist circumference; SBP, systolic blood pressure; DBP, Diastolic blood pressure; RHR, resting heart rate; TG, triglycerides; HDL, high-density lipoprotein; FBG, fasting blood glucose ^a^ Mean difference; ^b^ Standard mean difference.

## Supplementary Materials

The following supporting information can be downloaded at https://www.mdpi.com/article/10.3390/jcm13175221/s1, Method S1. Detailed search strategy; Results S1. Assessment of risk of bias of randomized trials with The Risk of Bias 2 (RoB2) and non-randomized trials with ROBINS-I; Results S2. Forest-plot showing the effect size (mean differences) of small-sided soccer games interventions vs. control groups; Results S4. Subgroup analyses on small-sided soccer games interventions vs. control group; Results S5. Leave-one-out analysis SSSG vs. control group; Results S6. Leave-one-out analysis sssg vs. other interventions.
