# Supplementary material for: Effects of Small-Sided Soccer Games on Physical Fitness and Cardiometabolic Health Biomarkers in Untrained Children and Adolescents: A Systematic Review and Meta-Analysis
Source: J Clin Med. 2024 Sep 3;13(17):5221. doi: 10.3390/jcm13175221 (PMC11396522; doi:10.3390/jcm13175221)
Supplement: Supplementary file 1 [file jcm-13-05221-s001.zip › jcm-3163395-supplementary.pdf]

SUPPLEMENTAL MATERIALS

sMethod S1. Detailed search strategy 2

sResults S1. Assessment of risk of bias of randomized trials with The Risk of Bias 2 (RoB2) and non-randomized trials with ROBINS-I. 7

sResults S2. Forest-Plot showing the effect size (Mean Differences) of small-sided soccer games interventions vs control groups. 8

Forest-Plot showing the effect size (Hedges g) of small-sided soccer games interventions vs Control Group on Physical Fitness between intervention and control groups for each study. 8

Forest-Plot showing the effect size (Mean Difference) of small-sided soccer games interventions on Anthropometric and body composition between intervention and control groups for each study. 9

sResults S3. Forest-Plot showing the effect size (Hedges g) of small-sided soccer games interventions vs other interventions 10

Forest-Plot showing the effect size (Hedges g) of small-sided soccer games interventions vs other intervention on Physical Fitness between intervention and control groups for each study. 10

Forest-Plot showing the effect size (Mean Difference) of small-sided soccer games interventions vs other intervention on Anthropometric and body composition between intervention and control groups for each study. 11

Forest-Plot showing the effect size (Mean Difference) of small-sided soccer games interventions vs other intervention on Cardiometabolic Variables between intervention and control groups for each study. 11

sResults S4. Results for subgroups SSSG vs Control Group 12

sResults S5. Leave-One-Out Analysis SSSG vs Control Group 13

Leave-One-Out Analysis for Physical Fitness **Error! Bookmark not defined.**

Leave-One-Out Analysis for Anthropometric and body composition 13

Leave-One-Out Analysis for Cardiometabolic Variables 15

sResults S6. Leave-One-Out Analysis SSSG vs other interventions 17

Leave-One-Out Analysis for Physical fitness 17

Leave-One-Out Analysis for Anthropometric and body composition 17

Leave-One-Out Analysis for Cardiovascular Variables 17

sMethod S1. Detailed search strategy

Supplementary table S1.1. Search terms for query

#1	Child OR Adolescent OR Childhood OR Children
#2	athletes OR “soccer player” OR “Soccer players”
#3	Soccer OR Small-sided Games OR Football OR “SSG”
#4	"Physical fitness” OR "Cardiorespiratory fitness" OR “physical performance” OR fitness
#5	“Metabolic syndrome” OR “Syndrome X” OR MetS
#6	“Waist circumference” OR WC OR "Body weight” OR "Body Mass Index" OR “body composition” OR anthropometry OR obesity OR overweight OR “body size” OR BMI OR “fat mass”
#7	Glucose OR blood glucose OR Hyperglycemia OR Insulin Resistance OR HOMA-IR OR HbA1c OR Hemoglobin A1c OR Diabetes Mellitus
#8	Cholesterol OR Triglycerides OR “Cholesterol, HDL” OR “Cholesterol, LDL”
#9	“Blood pressure” OR “systolic Pressure” OR “Diastolic Pressure” OR Hypertension OR SBP OR DBP
#10	Inflammation OR inflammatory OR “C-reactive protein” OR “CRP” OR “hsCRP” OR “IL-6” OR TNF-α
E1	“#1” AND “#3” AND “# 4” NOT “#2”
E2	“#1” AND “#3” AND “# 5” NOT “#2”
E3	“#1” AND “#3” AND “# 6” NOT “#2”
E4	“#1” AND “#3” AND “# 7” NOT “#2”
E5	“#1” AND “#3” AND “# 8” NOT “#2”
E6	“#1” AND “#3” AND “# 9” NOT “#2”
E7	“#1” AND “#3” AND “# 10” NOT “#2”

Supplementary table S1.2. Search terms for query

Database	strategy	Search strategy details
Web of Science	E1	Child OR Adolescent OR Childhood OR Children (Topic) AND Soccer OR Small-sided Games OR Football OR "SSG" (Topic) AND "Physical fitness" OR "Cardiorespiratory fitness" OR "physical performance" OR fitness (Topic) NOT athletes OR "soccer player" OR "Soccer players" (Topic)
	E2	Child OR Adolescent OR Childhood OR Children (Topic) AND Soccer OR Small-sided Games OR Football OR "SSG" (Topic) AND "Metabolic syndrome" OR "Syndrome X" OR MetS (Topic) NOT athletes OR "soccer player" OR "Soccer players" (Topic)
	E3	Child OR Adolescent OR Childhood OR Children (Topic) AND Soccer OR Small-sided Games OR Football OR "SSG" (Topic) AND "Waist circumference" OR WC OR "Body weight" OR "Body Mass Index" OR "body composition" OR anthropometry OR obesity OR overweight OR "body size" OR BMI OR "fat mass" (Topic) NOT athletes OR "soccer player" OR "Soccer players" (Topic)
	E4	Child OR Adolescent OR Childhood OR Children (Topic) AND Soccer OR Small-sided Games OR Football OR "SSG" (Topic) AND Glucose OR blood glucose OR Hyperglycemia OR Insulin Resistance OR HOMA-IR OR HbA1c OR Hemoglobin A1c OR Diabetes Mellitus (Topic) NOT athletes OR "soccer player" OR "Soccer players" (Topic)
	E5	Child OR Adolescent OR Childhood OR Children (Topic) AND Soccer OR Small-sided Games OR Football OR "SSG" (Topic) AND Cholesterol OR Triglycerides OR Cholesterol HDL OR Cholesterol LDL (Topic) NOT athletes OR "soccer player" OR "Soccer players" (Topic)
	E6	Child OR Adolescent OR Childhood OR Children (Topic) AND Soccer OR Small-sided Games OR Football OR "SSG" (Topic) AND "Blood pressure" OR "systolic Pressure" OR "Diastolic Pressure" OR Hypertension OR SBP OR DBP (Topic) NOT athletes OR "soccer player" OR "Soccer players" (Topic)
	E7	Child OR Adolescent OR Childhood OR Children (Topic) AND Soccer OR Small-sided Games OR Football OR "SSG" (Topic) AND Inflammation OR inflammatory OR "C-reactive protein" OR "CRP" OR "hsCRP" OR "IL-6" OR TNF-α (Topic) NOT athletes OR "soccer player" OR "Soccer players" (Topic)
Scopus	E1	(TITLE-ABS-KEY (child OR adolescent OR childhood OR children) AND TITLE-ABS-KEY (soccer OR small-sided AND games OR football OR ssg) AND TITLE-ABS-KEY ("Physical fitness" OR "Cardiorespiratory fitness" OR "physical performance" OR fitness) AND NOT TITLE-ABS-KEY (athletes OR "soccer player" OR "Soccer players"))
	E2	(TITLE-ABS-KEY (child OR adolescent OR childhood OR children) AND TITLE-ABS-KEY (soccer OR small-sided AND games OR football OR ssg) AND TITLE-ABS-KEY ("Metabolic syndrome" OR "Syndrome X" OR mets) AND NOT TITLE-ABS-KEY (athletes OR "soccer player" OR "Soccer players"))
	E3	(TITLE-ABS-KEY (child OR adolescent OR childhood OR children) AND TITLE-ABS-KEY (soccer OR small-sided AND games OR football OR ssg) AND TITLE-ABS-KEY ("Waist circumference" OR wc OR "Body weight" OR "Body Mass Index" OR "body composition" OR anthropometry OR obesity OR overweight OR "body size" OR bmi OR "fat mass") AND NOT TITLE-ABS-KEY (athletes OR "soccer player" OR "Soccer players"))
	E4	(TITLE-ABS-KEY (child OR adolescent OR childhood OR children) AND TITLE-ABS-KEY (soccer OR small-sided AND games OR football OR ssg) AND TITLE-ABS-KEY (glucose OR blood AND glucose OR hyperglycemia OR insulin AND resistance OR homa-ir OR hba1c OR hemoglobin AND a1c OR diabetes AND mellitus) AND NOT TITLE-ABS-KEY (athletes OR "soccer player" OR "Soccer players"))
	E5	(TITLE-ABS-KEY (child OR adolescent OR childhood OR children) AND TITLE-ABS-KEY (soccer OR small-sided AND games OR football OR ssg) AND TITLE-

		ABS-KEY (cholesterol OR triglycerides OR cholesterol AND hdl OR cholesterol AND ldl) AND NOT TITLE-ABS-KEY (athletes OR "soccer player" OR "Soccer players"))
	E6	(TITLE-ABS-KEY (child OR adolescent OR childhood OR children) AND TITLE-ABS-KEY (soccer OR small-sided AND games OR football OR ssg) AND TITLE-ABS-KEY ("Blood pressure" OR "systolic Pressure" OR "Diastolic Pressure" OR hypertension OR sbp OR dbp) AND NOT TITLE-ABS-KEY (athletes OR "soccer player" OR "Soccer players"))
	E7	(TITLE-ABS-KEY (child OR adolescent OR childhood OR children) AND TITLE-ABS-KEY (soccer OR small-sided AND games OR football OR ssg) AND TITLE-ABS-KEY (inflammation OR inflammatory OR "C-reactive protein" OR "CRP" OR "hsCRP" OR "IL-6" OR tnf-α) AND NOT TITLE-ABS-KEY (athletes OR "soccer player" OR "Soccer players"))
PubMed	E1	(("child"[Title/Abstract] OR "adolescent"[Title/Abstract] OR "childhood"[Title/Abstract] OR "children"[Title/Abstract]) AND ("soccer"[Title/Abstract] OR "small sided games"[Title/Abstract] OR "football"[Title/Abstract] OR "SSG"[Title/Abstract]) AND ("Physical fitness"[Title/Abstract] OR "Cardiorespiratory fitness"[Title/Abstract] OR "physical performance"[Title/Abstract] OR "fitness"[Title/Abstract])) NOT ("athletes"[Title/Abstract] OR "soccer player"[Title/Abstract] OR "Soccer players"[Title/Abstract])
	E2	(("child"[Title/Abstract] OR "adolescent"[Title/Abstract] OR "childhood"[Title/Abstract] OR "children"[Title/Abstract]) AND ("soccer"[Title/Abstract] OR "small sided games"[Title/Abstract] OR "football"[Title/Abstract] OR "SSG"[Title/Abstract]) AND ("Metabolic syndrome"[Title/Abstract] OR "Syndrome X"[Title/Abstract] OR "MetS"[Title/Abstract])) NOT ("athletes"[Title/Abstract] OR "soccer player"[Title/Abstract] OR "Soccer players"[Title/Abstract])
	E3	(("child"[Title/Abstract] OR "adolescent"[Title/Abstract] OR "childhood"[Title/Abstract] OR "children"[Title/Abstract]) AND ("soccer"[Title/Abstract] OR "small sided games"[Title/Abstract] OR "football"[Title/Abstract] OR "SSG"[Title/Abstract]) AND ("Waist circumference"[Title/Abstract] OR "WC"[Title/Abstract] OR "Body weight"[Title/Abstract] OR "Body Mass Index"[Title/Abstract] OR "body composition"[Title/Abstract] OR "anthropometry"[Title/Abstract] OR "obesity"[Title/Abstract] OR "overweight"[Title/Abstract] OR "body size"[Title/Abstract] OR "BMI"[Title/Abstract] OR "fat mass"[Title/Abstract])) NOT ("athletes"[Title/Abstract] OR "soccer player"[Title/Abstract] OR "Soccer players"[Title/Abstract])
	E4	(("child"[Title/Abstract] OR "adolescent"[Title/Abstract] OR "childhood"[Title/Abstract] OR "children"[Title/Abstract]) AND ("soccer"[Title/Abstract] OR "small sided games"[Title/Abstract] OR "football"[Title/Abstract] OR "SSG"[Title/Abstract]) AND ("Glucose"[Title/Abstract] OR "blood glucose"[Title/Abstract] OR "Hyperglycemia"[Title/Abstract] OR "insulin resistance"[Title/Abstract] OR "HOMA-IR"[Title/Abstract] OR "HbA1c"[Title/Abstract] OR "hemoglobin a1c"[Title/Abstract] OR "diabetes mellitus"[Title/Abstract])) NOT ("athletes"[Title/Abstract] OR "soccer player"[Title/Abstract] OR "Soccer players"[Title/Abstract])
	E5	(("child"[Title/Abstract] OR "adolescent"[Title/Abstract] OR "childhood"[Title/Abstract] OR "children"[Title/Abstract]) AND ("soccer"[Title/Abstract] OR "small sided games"[Title/Abstract] OR "football"[Title/Abstract] OR "SSG"[Title/Abstract]) AND ("Cholesterol"[Title/Abstract] OR "Triglycerides"[Title/Abstract] OR "cholesterol hdl"[Title/Abstract] OR "cholesterol ldl"[Title/Abstract])) NOT

		("athletes"[Title/Abstract] OR "soccer player"[Title/Abstract] OR "Soccer players"[Title/Abstract])
	E6	(("child"[Title/Abstract] OR "adolescent"[Title/Abstract] OR "childhood"[Title/Abstract] OR "children"[Title/Abstract]) AND ("soccer"[Title/Abstract] OR "small sided games"[Title/Abstract] OR "football"[Title/Abstract] OR "SSG"[Title/Abstract]) AND ("Blood pressure"[Title/Abstract] OR "systolic Pressure"[Title/Abstract] OR "Diastolic Pressure"[Title/Abstract] OR "Hypertension"[Title/Abstract] OR "SBP"[Title/Abstract] OR "DBP"[Title/Abstract])) NOT ("athletes"[Title/Abstract] OR "soccer player"[Title/Abstract] OR "Soccer players"[Title/Abstract])
	E7	(("child"[Title/Abstract] OR "adolescent"[Title/Abstract] OR "childhood"[Title/Abstract] OR "children"[Title/Abstract]) AND ("soccer"[Title/Abstract] OR "small sided games"[Title/Abstract] OR "football"[Title/Abstract] OR "SSG"[Title/Abstract]) AND (inflammation [Title/Abstract] OR inflammatory [Title/Abstract] OR "C-reactive protein" [Title/Abstract] OR "CRP" [Title/Abstract] OR "hsCRP" [Title/Abstract] OR "IL-6" [Title/Abstract] OR tnf-α [Title/Abstract]) NOT ("athletes"[Title/Abstract] OR "soccer player"[Title/Abstract] OR "Soccer players"[Title/Abstract])
Cochrane	E1	Child OR Adolescent OR Childhood OR Children in Title Abstract Keyword AND Soccer OR Small-sided Games OR Football OR “SSG” in Title Abstract Keyword AND "Physical fitness" OR "Cardiorespiratory fitness" OR “physical performance” OR fitness in Title Abstract Keyword NOT athletes OR “soccer player” OR “Soccer players” in Title Abstract Keyword - (Word variations have been searched)
	E2	Child OR Adolescent OR Childhood OR Children in Title Abstract Keyword AND Soccer OR Small-sided Games OR Football OR “SSG” in Title Abstract Keyword AND “Metabolic syndrome” OR “Syndrome X” OR MetS in Title Abstract Keyword NOT athletes OR “soccer player” OR “Soccer players” in Title Abstract Keyword - (Word variations have been searched)
	E3	Child OR Adolescent OR Childhood OR Children in Title Abstract Keyword AND Soccer OR Small-sided Games OR Football OR “SSG” in Title Abstract Keyword AND “Waist circumference" OR WC OR "Body weight” OR "Body Mass Index" OR “body composition” OR anthropometry OR obesity OR overweight OR “body size” OR BMI OR “fat mass” in Title Abstract Keyword NOT athletes OR “soccer player” OR “Soccer players” in Title Abstract Keyword - (Word variations have been searched)
	E4	Child OR Adolescent OR Childhood OR Children in Title Abstract Keyword AND Soccer OR Small-sided Games OR Football OR “SSG” in Title Abstract Keyword AND Glucose OR blood glucose OR Hyperglycemia OR Insulin Resistance OR HOMA-IR OR HbA1c OR Hemoglobin A1c OR Diabetes Mellitus in Title Abstract Keyword NOT athletes OR “soccer player” OR “Soccer players” in Title Abstract Keyword - (Word variations have been searched)
	E5	Child OR Adolescent OR Childhood OR Children in Title Abstract Keyword AND Soccer OR Small-sided Games OR Football OR “SSG” in Title Abstract Keyword AND Cholesterol OR Triglycerides OR Cholesterol HDL OR Cholesterol LDL in Title Abstract Keyword NOT athletes OR “soccer player” OR “Soccer players” in Title Abstract Keyword - (Word variations have been searched)
	E6	Child OR Adolescent OR Childhood OR Children in Title Abstract Keyword AND Soccer OR Small-sided Games OR Football OR “SSG” in Title Abstract Keyword AND “Blood pressure” OR “systolic Pressure” OR “Diastolic Pressure” OR Hypertension OR SBP OR DBP in Title Abstract Keyword NOT athletes OR “soccer player” OR “Soccer players” in Title Abstract Keyword - (Word variations have been searched)
	E7	Child OR Adolescent OR Childhood OR Children in Title Abstract Keyword AND Soccer OR Small-sided Games OR Football OR “SSG” in Title Abstract Keyword AND Cholesterol OR Triglycerides OR Cholesterol HDL OR Cholesterol LDL in Title Abstract Keyword AND Inflammation OR inflammatory OR “C-reactive

		protein" OR "CRP" OR "hsCRP" OR "IL-6" OR TNF-α in Title Abstract Keyword NOT athletes OR "soccer player" OR "Soccer players" in Title Abstract Keyword - (Word variations have been searched)
EBSCO	E1	AB (Child OR Adolescent OR Childhood OR Children) AND AB (Soccer OR Small-sided Games OR Football OR "SSG") AND ("Physical fitness" OR "Cardiorespiratory fitness" OR "physical performance" OR fitness) NOT (athletes OR "soccer player" OR "Soccer players")
	E2	AB (Child OR Adolescent OR Childhood OR Children) AND AB (Soccer OR Small-sided Games OR Football OR "SSG") AND AB ("Metabolic syndrome" OR "Syndrome X" OR MetS) NOT AB (athletes OR "soccer player" OR "Soccer players")
	E3	AB (Child OR Adolescent OR Childhood OR Children) AND AB (Soccer OR Small-sided Games OR Football OR "SSG") AND AB ("Waist circumference" OR WC OR "Body weight" OR "Body Mass Index" OR "body composition" OR anthropometry OR obesity OR overweight OR "body size" OR BMI OR "fat mass") NOT AB (athletes OR "soccer player" OR "Soccer players")
	E4	AB (Child OR Adolescent OR Childhood OR Children) AND AB (Soccer OR Small-sided Games OR Football OR "SSG") AND AB (Glucose OR blood glucose OR Hyperglycemia OR Insulin Resistance OR HOMA-IR OR HbA1c OR Hemoglobin A1c OR Diabetes Mellitus) NOT AB (athletes OR "soccer player" OR "Soccer players")
	E5	AB (Child OR Adolescent OR Childhood OR Children) AND AB (Soccer OR Small-sided Games OR Football OR "SSG") AND AB (Cholesterol OR Triglycerides OR Cholesterol HDL OR Cholesterol LDL) NOT AB (athletes OR "soccer player" OR "Soccer players")
	E6	AB (Child OR Adolescent OR Childhood OR Children) AND AB (Soccer OR Small-sided Games OR Football OR "SSG) AND AB ("Blood pressure" OR "systolic Pressure" OR "Diastolic Pressure" OR Hypertension OR SBP OR DBP) NOT AB (athletes OR "soccer player" OR "Soccer players")
	E7	AB (Child OR Adolescent OR Childhood OR Children) AND AB (Soccer OR Small-sided Games OR Football OR "SSG") AND AB (Inflammation OR inflammatory OR "C-reactive protein" OR "CRP" OR "hsCRP" OR "IL-6" OR TNF-α) NOT AB (athletes OR "soccer player" OR "Soccer players")

sResults S1. Assessment of risk of bias of randomized trials with The Risk of Bias 2 (RoB2) and non-randomized trials with ROBINS-I.

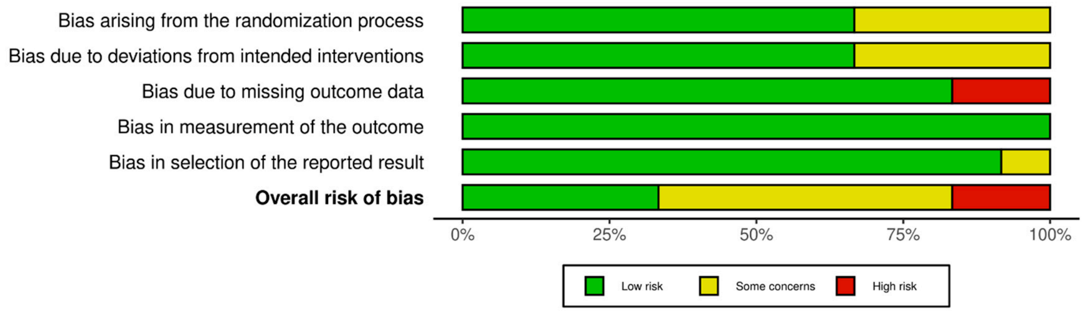

Supplementary Figure S1. Assessment of risk of bias of randomized trials with The Risk of Bias 2 (RoB2)

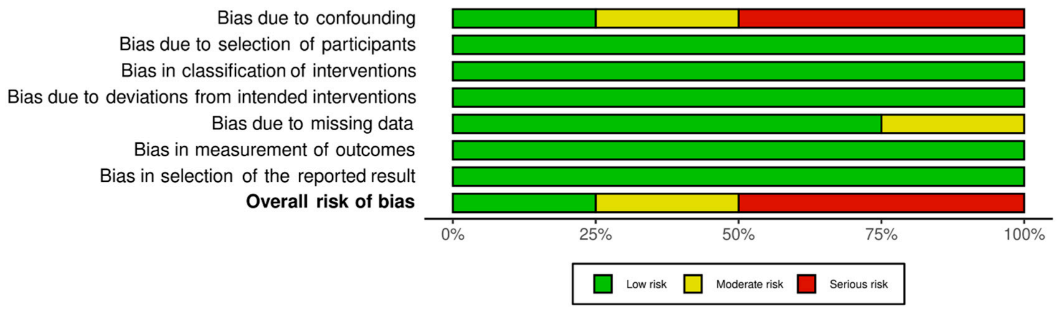

Supplementary Figure S2. Assessment of risk of bias of non-randomized trials with ROBINS-I.

sResults S2. Forest-plot showing the effect size (mean differences) of small-sided soccer games interventions vs control groups.

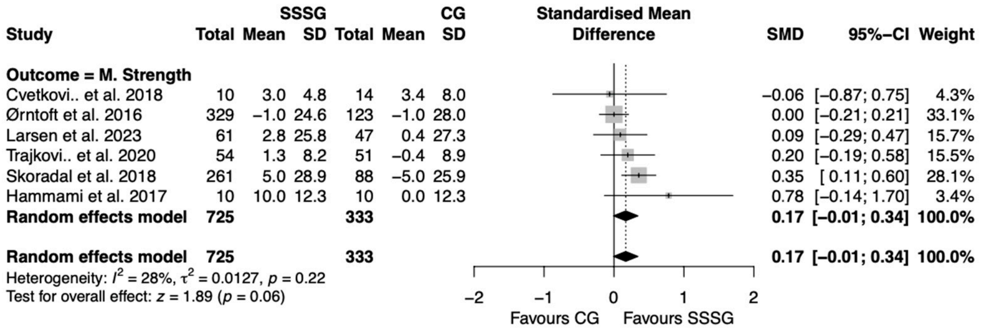

Supplementary Figure S3. Forest-Plot showing the effect size (Hedges g) of small-sided soccer games interventions vs Control Group on Physical Fitness between intervention and control groups for each study.

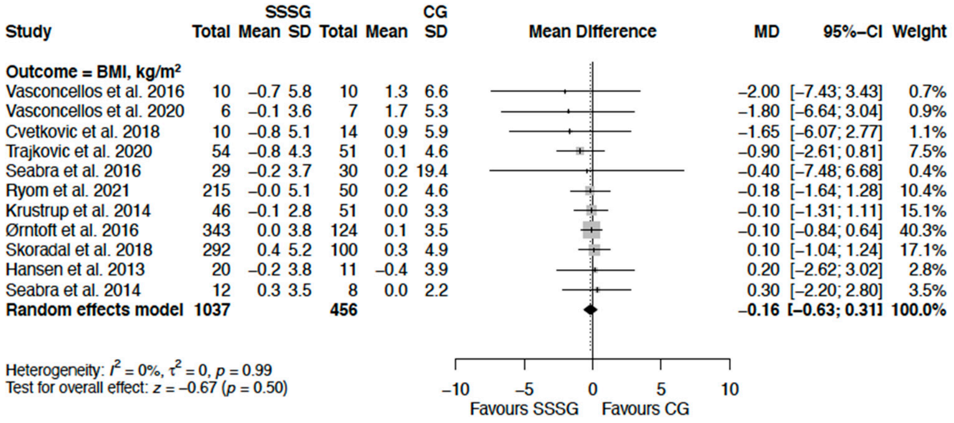

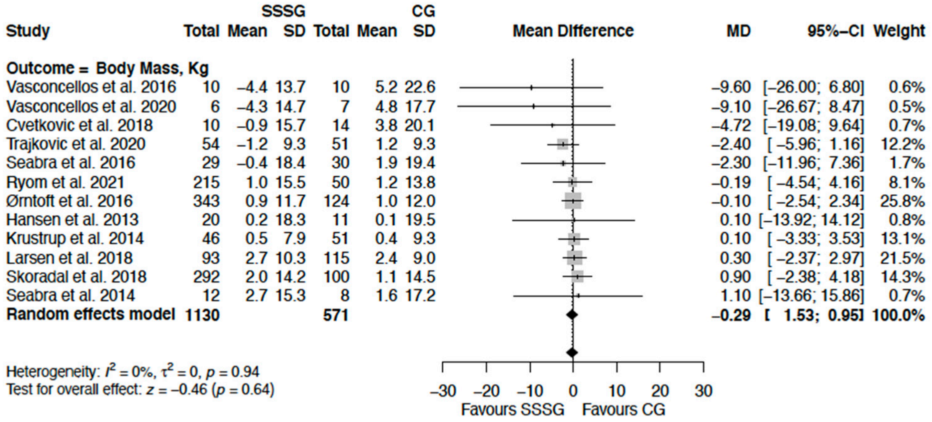

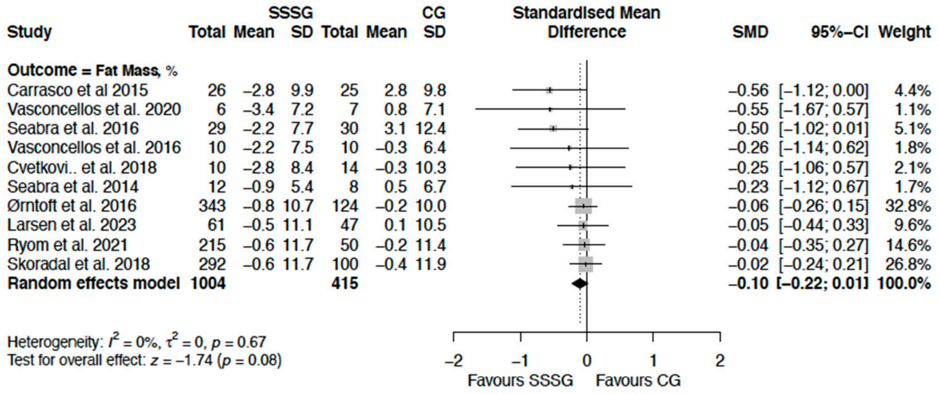

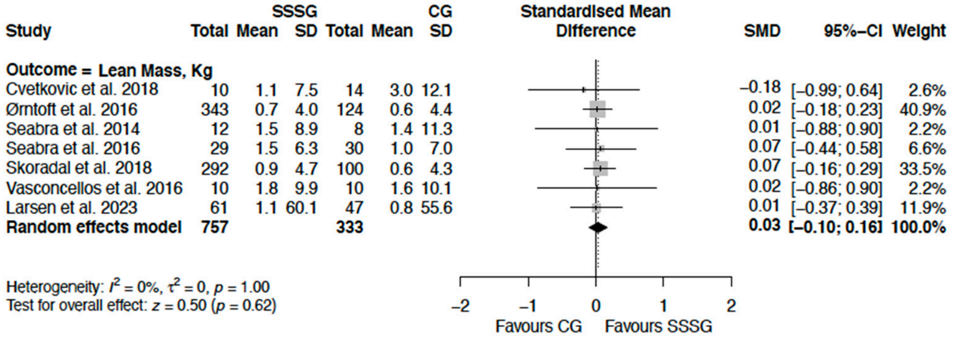

Supplementary Figure S4. Forest-Plot showing the effect size (Mean Difference) of small-sided soccer games interventions on Anthropometric and body composition between intervention and control groups for each study.

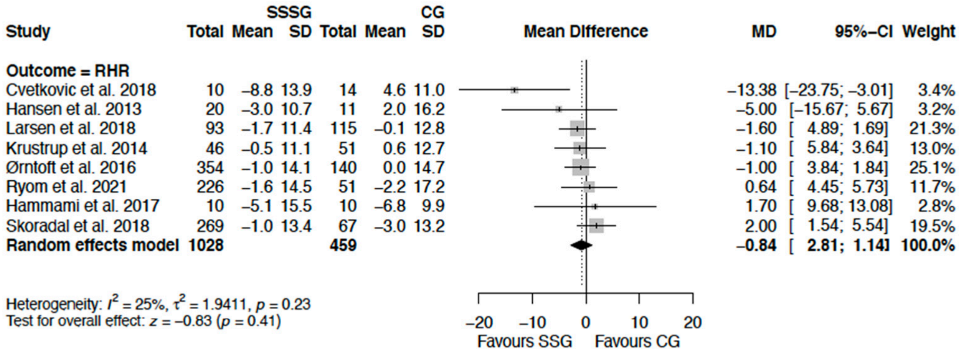

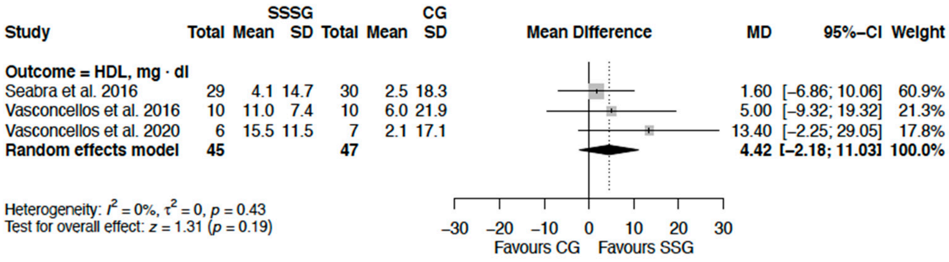

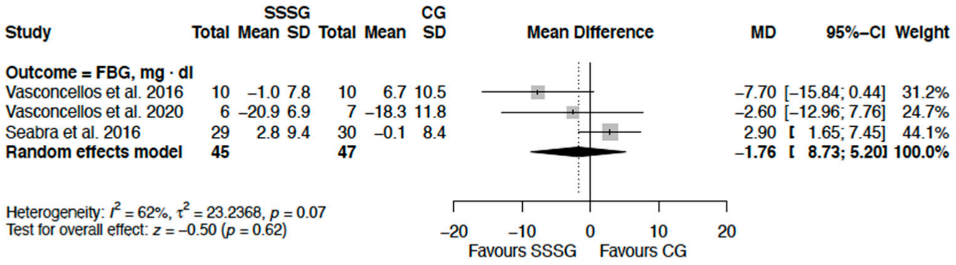

Supplementary Figure S5. Forest-Plot showing the effect size (Mean Difference) of small-sided soccer games interventions on Cardiometabolic Variables between intervention and control groups for each study.

sResults S3. Forest-plot showing the effect size (hedges g) of small-sided soccer games interventions vs other interventions.

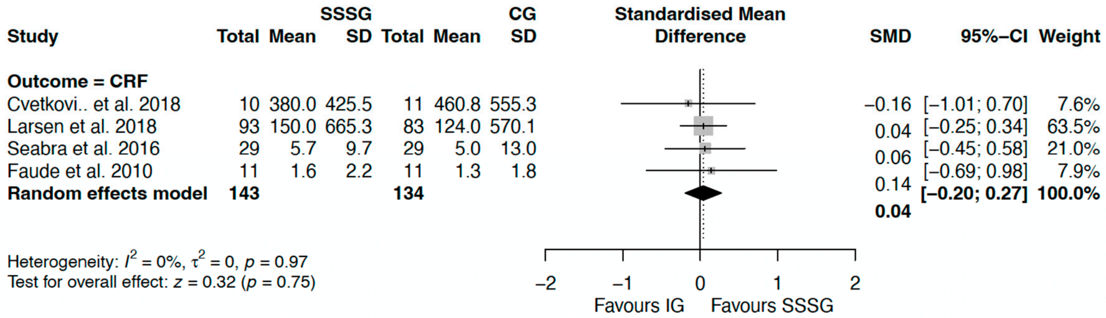

Supplementary Figure S6. Forest-Plot showing the effect size (Hedges g) of small-sided soccer games interventions vs other intervention in Cardiorespiratory fitness between intervention and control groups for each study.

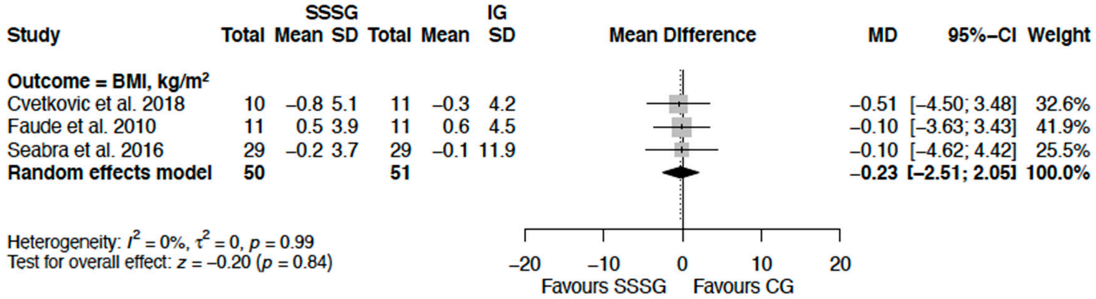

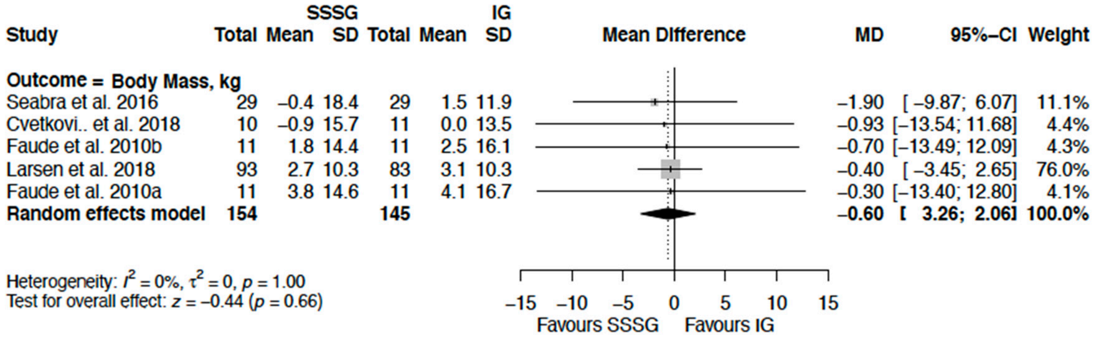

Supplementary Figure S7. Forest-Plot showing the effect size (Mean Difference) of small-sided soccer games interventions vs other intervention on Anthropometric and body composition between intervention and control groups for each study.

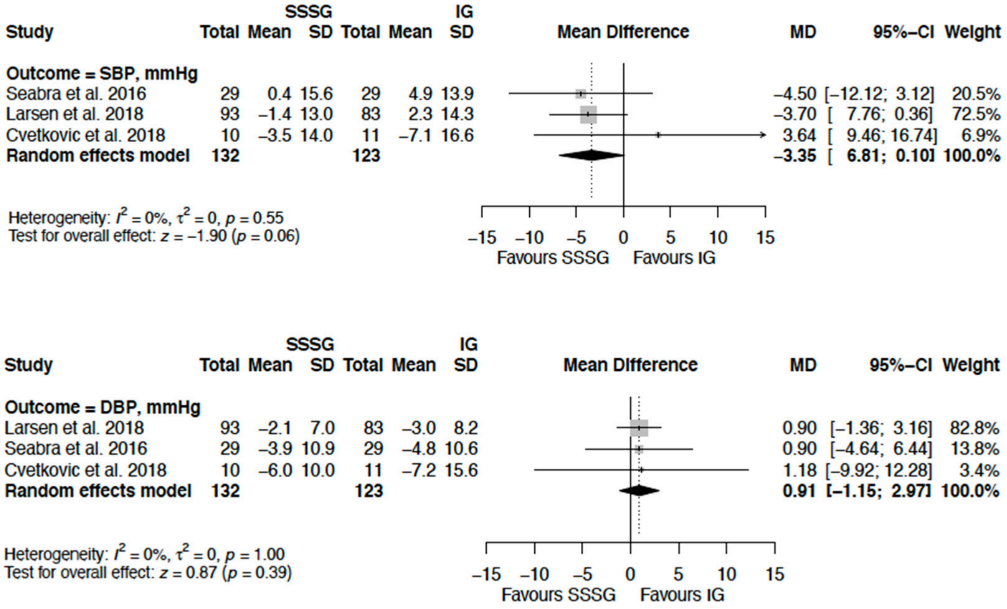

Supplementary Figure S8. Forest-Plot showing the effect size (Mean Difference) of small-sided soccer games interventions vs other intervention on Cardiometabolic Variables between intervention and control groups for each study.

sResults S4. Subgroup analyses on small-sided soccer games interventions vs control group.

Supplementary table S4.1. Results for subgroups SSSG vs Control Group

		k	SMD/MD [95%-CI]	I^2	p-value	k	MD [95%-CI]	I^2	p-value
Systolic Blood Pressure (MD)						***Diastolic Blood Pressure (MD)***			
Age	≥12 years	4	-6.07 [-9.26; -2.88]	0.0%	0.31	4	-2.68 [-5.70; 0.35]	0.0%	0.32
	< 12 years	7	-3.35 [-5.70; -1.01]	53.1 %		7	-1.00 [-2.44; 0.44]	39.6%	
Sex	Male	3	-1.62 [-7.26; 4.03]	0.0%	0.31	3	-4.91 [-8.85; -0.96]	0.0%	0.06
	Male-Female	8	-4.12 [-6.33; -1.90]	54.6 %		8	-1.01 [-2.08; 0.07]	8.9%	
Status	OW-O	5	-6.77 [-9.67; -3.87]	0.0%	0.16	5	-2.75 [-6.09; 0.59]	25.6%	0.60
	Healthy	1	-0.80 [-10.50; 8.90]	--		1	-1.70 [-8.74; 5.34]	--	
	All	5	-2.90 [-5.07; -0.72]	47.1 %		5	-0.94 [-2.25; 0.36]	31.5%	
Duration (weeks)	<20 weeks	9	-4.23 [-6.53; -1.94]	47.0 %	0.17	9	-0.87 [-2.09; 0.36]	12.4%	0.15
	≥20 weeks	2	-2.26 [-5.57; 1.04]	0.0%		2	-2.98 [-5.64; -0.31]	12.6%	
Design	RCT	4	-6.07 [-9.27; -2.88]	0.0%	0.42	4	-2.68 [-5.70; 0.35]	0.0%	0.59
	Non-RCT	2	-6.62 [-17.40; 4.15]	72.5 %		2	-1.25 [-10.72; 8.23]	73.7%	
	cRCT	5	-2.90 [-5.07; -0.72]	47.1 %		5	-0.94 [-2.25; 0.36]	31.5%	
Cardiorespiratory Fitness (SMD)						***Muscular fitness (SMD)***			
Age	≥12 years	5	0.29 [0.03; 0.61]	0.0%	0.18	3	0.23 [-0.10; 0.55]	0.0%	0.70
	< 12 years	4	0.07 [0.09; 0.18]	0.0%		3	0.15 [-0.09; 0.39]	54.4%	
Sex	Male	4	0.32 [0.28; 0.92]	0.0%	0.43	2	0.32 [-0.28; 0.92]	0.0%	0.43
	Male-Female	5	0.09 [-0.03; 0.21]	0.0%		4	0.07 [-0.06; 0.20]	0.0%	
Status	OW-O	3	0.39 [0.43; 1.21]	--	0.72	1	0.39 [-0.43; 1.21]	--	0.72
	Healthy	1	0.24 [0.64; 1.12]	--		1	0.24 [-0.64; 1.12]	--	
	All	5	0.09 [-0.03; 0.21]	0.0%		4	0.07 [-0.06; 0.20]	0.0%	
Duration (weeks)	<20 weeks	6	0.10 [0.04; 0.23]	0.0%	0.73	4	0.07 [-0.08; 0.22]	0.0%	0.73
	≥20 weeks	3	0.12 [0.11; 0.34]	2.5%		2	0.12 [-0.11; 0.34]	2.5%	
Design	RCT	4	0.32 [0.28; 0.92]	0.0%	0.43	2	0.33 [-0.49; 1.16]	45.3%	0.72
	cRCT	5	0.09 [-0.03; 0.21]	0.0%		3	0.17 [-0.06; 0.41]	58.2%	

sResults S5. Leave-one-out analysis SSSG vs control group

Leave-One-Out Analysis for health-related physical fitness

Supplementary table S5.1. Cardiorespiratory Fitness Leave-One-Out Analysis

	Leave-One-Out Analysis (Sorted by I2)			Influence Diagnostics							
	Effect	IC 95%	I^2	rstudent	dffits	cook.d	cov.r	QE.del	hat	weight	Infl
Omitting Cvetković et al. 2018	0.08	-0.05; 0.20	0	0.73	0.11	0.01	1.02	1.43	0.02	2.35	N
Omitting Hammami et al. 2017	0.08	-0.05; 0.21	0	0.36	0.05	0.00	1.02	1.83	0.02	2.04	N
Omitting Larsen et al. 2018	0.10	-0.04; 0.24	0	-0.40	-0.21	0.04	1.27	1.80	0.21	21.13	N
Omitting Ørntoft et al. 2016	0.09	-0.06; 0.25	0	-0.21	-0.16	0.03	1.582	1.92	0.37	36.78	N
Omitting Seabra et al. 2016	0.12	0.00; 0.23	0	0.51	0.12	0.01	1.05	5.75	0.05	4.84	N
Omitting Skoradal et al. 2018	0.10	-0.04; 0.25	0	-0.50	-0.30	0.10	1.37	1.71	0.27	27.04	N
Omitting Trajković et al. 2020	0.06	-0.07; 0.19	0	1.05	0.36	0.13	1.12	0.86	0.11	10.67	N
Omitting Vasconcellos et al. 2016	0.11	-0.01; 0.2	0	1.79	0.22	0.05	1.02	2.81	0.01	1.45	N
Omitting Ryom et al. 2021	0.11	-0.01; 0.2	0	0.60	0.23	0.05	1.15	5.65	0.13	13.14	N

rstudent, Externally Standardized Residuals; dffits, DFFITS Value; cook.d, Cook's Distance; cov.r, Covariance Ratio; QE.del, Leave-One-Out τ2 and Q Values; hat, Hat Value; weight, Study Weight; Infl, Influence.

Supplementary table S5.2. Muscular strength leave-One-Out Analysis

	Leave-One-Out Analysis (Sorted by I2)			Influence Diagnostics							
	Effect	IC 95%	I2	rstudent	dffits	cook.d	cov.r	QE.del	hat	weight	Infl
Omitting Ørntoft et al. 2016	0.31	0.11; 0.50	0	-2.13	-1.88	1.15	0.80	2.29	0.36	36.17	Y
Omitting Skoradal et al. 2018	0.08	-0.12; 0.28	8	1.57	1.36	0.97	0.84	3.27	0.32	32.20	Y
Omitting Hammami et al. 2017	0.16	-0.05; 0.37	41	1.25	0.29	0.08	0.92	5.05	0.05	5.11	N
Omitting Cvetković et al. 2018	0.21	-0.03; 0.46	54	-0.59	-0.21	0.05	1.26	6.53	0.06	6.36	N
Omitting Trajković et al. 2020	0.20	-0.10; 0.49	56	-0.01	-0.07	0.01	1.81	6.80	0.20	20.16	N
Omitting Larsen et al. 2023	0.19	-0.03 0.41	42	-0.37	-0.22	0.06	1.57	6.84	0.16	15.66	N

rstudent, Externally Standardized Residuals; dffits, DFFITS Value; cook.d, Cook's Distance; cov.r, Covariance Ratio; QE.del, Leave-One-Out τ2 and Q Values; hat, Hat Value; weight, Study Weight; Infl, Influence.

Leave-One-Out Analysis for Anthropometric and body composition

Supplementary table S5.3. Body Mass Index (BMI) leave-One-Out Analysis

	Leave-One-Out Analysis (Sorted by I2)		Influence Diagnostics							
	Effect [IC 95%]	I2	rstudent	dffits	cook.d	cov.r	QE.del	hat	weight	Infl
Omitting Cvetković et al. 2018	-0.14 [-0.64; 0.36]	0	-0.67	-0.08	0.01	1.01	2.02	0.01	1.26	N
Omitting Hansen et al. 2013	-0.17 [-0.68; 0.33]	0	0.25	0.05	0.00	1.03	2.40	0.03	3.09	N
Omitting Krustrup et al. 2014	-0.17 [-0.72; 0.37]	0	0.10	0.05	0.00	1.20	2.45	0.17	16.89	N
Omitting Ørntoft et al. 2016	-0.21 [-0.88; 0.46]	0	0.21	0.19	0.05	1.82	2.42	0.45	44.99	Y
Omitting Seabra et al. 2014	-0.18 [-0.68; 0.33]	0	0.37	0.07	0.01	1.04	2.33	0.04	3.94	N
Omitting Seabra et al. 2016	-0.16 [-0.66; 0.34]	0	-0.07	-0.01	0.00	1.01	2.46	0.01	0.49	N
Omitting Skoradal et al. 2018	-0.22 [-0.77; 0.33]	0	0.50	0.24	0.06	1.24	2.22	0.19	19.06	N
Omitting Trajković et al. 2020	-0.09 [-0.61; 0.43]	0	-0.89	-0.27	0.07	1.09	1.68	0.08	8.39	N

Omitting Vasconcellos et al. 2016	-0.14 [-0.64; 0.36]	0	-0.67	-0.06	0.00	1.01	2.02	0.01	0.84	N
Omitting Vasconcellos et al. 2020	-0.14 [-0.64; 0.36]	0	-0.67	-0.07	0.01	1.01	2.02	0.01	1.05	N
Omitting Ryom et al. 2021	-0.16 [-0.66; 0.34]	0	-0.03	-0.01	0.00	1.12	2.47	0.10	10.38	N

rstudent, Externally Standardized Residuals; dffits, DFFITS Value; cook.d, Cook's Distance; cov.r, Covariance Ratio; QE.del, Leave-One-Out τ2 and Q Values; hat, Hat Value; weight, Study Weight; Infl, Influence.

Supplementary table S5.4. Body mass Leave-One-Out Analysis

	Leave-One-Out Analysis (Sorted by I2)		Influence Diagnostics							
	Effect [IC 95%]	I2	rstudent	dffits	cook.d	cov.r	QE.del	hat	weight	Infl
Omitting Cvetković et al. 2018	-0.27 [-1.57; 1.04]	0	-0.61	-0.06	0.00	1.01	4.52	0.01	0.81	N
Omitting Hansen et al. 2013	-0.30 [-1.61; 0.99]	0	0.06	0.01	0.00	1.01	4.89	0.01	0.85	N
Omitting Krustrup et al. 2014	-0.37 [-1.77; 1.03]	0.	0.25	0.10	0.01	1.17	4.83	0.14	14.25	N
Omitting Larsen et al. 2018	-0.49 [-1.97; 0.99]	0	0.50	0.28	0.08	1.31	4.64	0.24	23.45	N
Omitting Ørntoft et al. 2016	-0.38 [-1.91; 1.15]	0	0.19	0.12	0.01	1.39	4.85	0.28	28.06	Y
Omitting Seabra et al. 2014	-0.31 [-1.61; 0.99]	0	0.19	0.02	0.00	1.01	4.86	0.01	0.77	N
Omitting Seabra et al. 2016	-0.26 [-1.57; 1.04]	0	-0.41	-0.06	0.00	1.02	4.72	0.02	1.80	N
Omitting Skoradal et al. 2018	-0.52 [-1.93; 0.89]	0	0.78	0.34	0.11	1.19	4.28	0.16	15.58	N
Omitting Trajković et al. 2020	0.02 [-1.37; 1.41]	0	-1.24	-0.49	0.24	1.15	3.35	0.13	13.26	N
Omitting Vasconcellos et al. 2016	-0.24 [-1.54; 1.06]	0	-1.12	-0.09	0.02	1.01	3.65	0.01	0.62	N
Omitting Vasconcellos et al. 2020	-0.25 [-1.55; 1.05]	0	-0.98	-0.07	0.01	1.01	3.92	0.01	0.54	N
Omitting Ryom et al. 2021	-0.30 [-1.60; 0.99]	0	0.05	0.01	0.00	1.09	4.89	0.08	8.15	N

rstudent, Externally Standardized Residuals; dffits, DFFITS Value; cook.d, Cook's Distance; cov.r, Covariance Ratio; QE.del, Leave-One-Out τ2 and Q Values; hat, Hat Value; weight, Study Weight; Infl, Influence.

Supplementary table S5.5 %Fat Mass Leave-One-Out Analysis

	Leave-One-Out Analysis (Sorted by I2)		Influence Diagnostics							
	Effect [IC 95%]	I2	rstudent	dffits	cook.d	cov.r	QE.del	hat	weight	Infl
Omitting Ørntoft et al. 2016	-0.13 [-0.27; 0.02]	0	0.84	0.73	0.54	1.76	5.70	0.43	43.24	Y
Omitting Seabra et al. 2016	-0.10 [-0.24; 0.05]	0	-1.49	-0.40	0.16	1.07	4.18	0.07	6.77	N
Omitting Skoradal et al. 2018	-0.18 [-0.35; -0.01]	0	1.14	0.84	0.71	1.55	5.11	0.35	35.37	N
Omitting Vasconcellos et al. 2020	-0.12 [-0.25; 0.02]	0	-0.75	-0.09	0.01	1.02	5.84	0.02	1.46	N
Omitting Carrasco et al 2015	-0.10 [-0.24; 0.04]	0	-1.56	-0.39	0.15	1.06	3.96	0.06	5.81	N
Omitting Vasconcellos et al. 2016	-0.13 [-0.28; 0.02]	0	-0.29	0.09	0.01	1.18	6.31	0.02	2.35	N
Omitting Cvetković et al. 2018	-0.13 [-0.28; 0.02]	0	-0.27	0.10	0.01	1.19	6.32	0.03	2.75	N
Omitting Seabra et al. 2014	-0.13 [-0.28; 0.02]	0	0.13	0.02	1.20	6.35	0.02	2.26	-0.20	N
Omitting Larsen et al. 2023	-0.11 [-0.23; 0.01]	0	0.27	0.09	0.01	1.11	6.64	0.10	9.56	N
Omitting Ryom et al. 2021	-0.11 [-0.24; 0.01]	0	0.44	0.18	0.03	1.17	6.52	0.15	14.61	N

rstudent, Externally Standardized Residuals; dffits, DFFITS Value; cook.d, Cook's Distance; cov.r, Covariance Ratio; QE.del, Leave-One-Out τ2 and Q Values; hat, Hat Value; weight, Study Weight; Infl, Influence.

Supplementary table S5.6. Lean Mass Leave-One-Out Analysis

	Leave-One-Out Analysis (Sorted by I2)		Influence Diagnostics							
	Effect [IC 95%]	I2	rstudent	dffits	cook.d	cov.r	QE.del	hat	weight	Infl
Omitting Cvetković et al. 2018	0.04 [-0.10; 0.19]	0	-0.52	-0.09	0.01	1.03	0.09	0.03	2.97	N
Omitting Ørntoft et al. 2016	0.05 [-0.14; 0.24]	0	-0.17	-0.16	0.03	1.87	0.34	0.47	46.50	N
Omitting Seabra et al. 2014	0.04 [-0.10; 0.18]	0	-0.06	-0.01	0.00	1.03	0.36	0.03	2.45	N
Omitting Seabra et al. 2016	0.03 [-0.11; 0.18]	0	0.15	0.04	0.00	1.08	0.34	0.08	7.52	N
Omitting Skoradal et al. 2018	0.02 [-0.16; 0.20]	0	0.31	0.24	0.06	1.61	0.27	0.38	38.02	N
Omitting Vasconcellos et al. 2016	0.04 [-0.10; 0.18]	0	-0.04	-0.01	0.00	1.03	0.36	0.03	2.55	N

Omitting Larsen et al. 2023	0.04 [-0.10; 0.18]	o	-0.15	-0.06	0.00	1.14	0.37	0.12	11.94	N

rstudent, Externally Standardized Residuals; dffits, DFFITS Value; cook.d, Cook's Distance; cov.r, Covariance Ratio; QE.del, Leave-One-Out τ2 and Q Values; hat, Hat Value; weight, Study Weight; Infl, Influence.

Supplementary table S5.7. Waist Circumference Leave-One-Out Analysis

	**Leave-One-Out Analysis (Sorted by I2)**		**Influence Diagnostics**							
	Effect [IC 95%]	I2	rstudent	dffits	cook.d	cov.r	QE.del	hat	weight	Infl
Omitting Vasconcellos et al. 2020	-4.57 [-11.15; 2.00]	0	-1.59	-0.83	0.57	0.76	0.10	0.20	20.38	Y
Omitting Seabra et al. 2016	-11.92 [-23.16; -0.68]	16	1.04	1.19	1.33	2.22	1.18	0.56	56.07	Y
Omitting Vasconcellos et al. 2016	-9.41 [-22.84; 4.03]	62	0.22	0.37	0.25	3.17	2.64	0.25	23.56	N

rstudent, Externally Standardized Residuals; dffits, DFFITS Value; cook.d, Cook's Distance; cov.r, Covariance Ratio; QE.del, Leave-One-Out τ2 and Q Values; hat, Hat Value; weight, Study Weight; Infl, Influence.

Leave-One-Out Analysis for Cardiometabolic Variables

Supplementary table S5.8. Systolic Blood Pressure Leave-One-Out Analysis

	**Leave-One-Out Analysis (Sorted by I2)**		**Influence Diagnostics**							
	Effect [IC 95%]	I2	rstudent	dffits	cook.d	cov.r	QE.del	hat	weight	Infl
Omitting Hansen et al. 2013	-4.15 [-5.60; -2.70]	0	-1.97	-0.30	0.08	0.66	7.96	0.05	4.84	N
Omitting Krustrup et al. 2014	-4.86 [-6.49; -3.22]	9	1.68	0.54	0.25	0.84	8.77	0.11	10.90	N
Omitting Vasconcellos et al. 2020	-3.95 [-5.79; -2.10]	18	-1.26	-0.53	0.26	1.07	9.70	0.15	15.18	N
Omitting Larsen et al. 2018	-4.76 [-6.74; -2.78]	24	0.89	0.38	0.15	1.23	10.57	0.16	15.87	N
Omitting Skoradal et al. 2018	-4.05 [-6.10; -2.01]	25	-0.79	-0.37	0.15	1.32	10.70	0.18	18.29	N
Omitting Seabra et al. 2016	-4.56 [-6.43; -2.69]	29	0.78	0.17	0.03	1.10	11.19	0.04	4.17	N
Omitting Hammami et al. 2017	-4.536 [-6.41; -2.67]	29	0.74	0.14	0.02	1.10	11.26	0.03	3.43	N
Omitting Ørntoft et al. 2016	-4.50 [-6.75; -2.24]	32	0.20	0.09	0.01	1.60	11.72	0.23	22.73	N
Omitting Vasconcellos et al. 2016	-4.35 [-6.27; -2.44]	32	-0.28	-0.06	0.00	1.15	11.77	0.03	2.68	N
Omitting Cvetković et al. 2018	-4.40 [-6.31; -2.49]	33	-0.00	-0.01	0.00	1.15	11.86	0.02	1.92	N
Omitting Ryom et al. 2021	-3.81 [-6.15 -1.48]	23	1.78	0.66	0.34	0.87	11.81	0.12	12.01	N

rstudent, Externally Standardized Residuals; dffits, DFFITS Value; cook.d, Cook's Distance; cov.r, Covariance Ratio; QE.del, Leave-One-Out τ2 and Q Values; hat, Hat Value; weight, Study Weight; Infl, Influence.

Supplementary table S5.9. Diastolic Blood Pressure Leave-One-Out Analysis

	**Leave-One-Out Analysis (Sorted by I2)**		**Influence Diagnostics**							
	Effect [IC 95%]	I2	rstudent	dffits	cook.d	cov.r	QE.del	hat	weight	Infl
Omitting Skoradal et al. 2018	-1.77 [-2.97; -0.57]	6	1.83	0.79	0.42	0.79	8.53	0.17	16.71	N
Omitting Seabra et al. 2016	-1.11 [-2.39; 0.17]	21	-1.47	-0.32	0.10	0.90	10.06	0.05	4.91	N
Omitting Cvetković et al. 2018	-1.18 [-2.44; 0.08]	21	-1.49	-0.22	0.05	0.87	10.09	0.02	2.34	N
Omitting Krustrup et al. 2014	-1.64 [-3.09; -0.19]	24	1.08	0.46	0.21	1.15	10.54	0.15	15.45	N
Omitting Hansen et al. 2013	-1.46 [-2.76; -0.15]	24	1.30	0.19	0.04	0.94	10.57	0.03	2.74	N
Omitting Ørntoft et al. 2016	-1.16 [-2.85; 0.54]	30	-0.47	-0.21	0.06	1.58	11.38	0.26	25.60	N
Omitting Larsen et al. 2018	-1.14 [-2.74; 0.45]	31	-0.59	-0.24	0.07	1.39	11.66	0.17	17.40	N
Omitting Vasconcellos et al. 2020	-1.27 [-2.79; 0.25]	35	-0.29	-0.07	0.01	1.27	12.25	0.09	8.90	N
Omitting Hammami et al. 2017	-1.32 [-2.79; 0.14]	35	-0.10	-0.00	0.00	1.18	12.35	0.04	3.68	N
Omitting Vasconcellos et al. 2016	-1.32 [-2.77; 0.13]	35	-0.15	-0.01	0.00	1.15	12.34	0.02	2.28	N
Omitting Ryom et al. 2021	-1.32 [-2.68; 0.03]	27	0.23	0.10	0.01	1.32	12.36	0.11	10.97	N

rstudent, Externally Standardized Residuals; dffits, DFFITS Value; cook.d, Cook's Distance; cov.r, Covariance Ratio; QE.del, Leave-One-Out τ2 and Q Values; hat, Hat Value; weight, Study Weight; Infl, Influence.

Supplementary table S5.10. Resting Heart rate Leave-One-Out Analysis

	**Leave-One-Out Analysis (Sorted by I2)**		**Influence Diagnostics**							
	Effect [IC 95%]	I2	rstudent	dffits	cook.d	cov.r	QE.del	hat	weight	Infl
Omitting Cvetković et al. 2018	-0.54 [-2.21; 1.14]	0	-2.40	-0.52	0.24	0.54	3.35	0.04	4.37	N
Omitting Skoradal et al. 2018	-1.81 [-4.01; 0.39]	15	1.60	0.71	0.36	0.94	5.88	0.22	21.86	N
Omitting Hansen et al. 2013	-0.97 [-3.39; 1.45]	41	-0.69	-0.12	0.01	1.13	8.50	0.04	4.14	N
Omitting Larsen et al. 2018	-1.18 [-4.19; 1.83]	43	-0.13	0.05	0.00	1.75	8.83	0.24	23.58	N
Omitting Hammami et al. 2017	-1.29 [-3.77; 1.18]	44	0.48	0.16	0.03	1.18	8.89	0.04	3.68	N
Omitting Krustrup et al. 2014	-1.26 [-4.05; 1.53]	45	0.05	0.12	0.02	1.51	9.08	0.15	15.42	N
Omitting Ørntoft et al. 2016	-1.43 [-4.60; 1.74]	45	0.13	0.21	0.08	1.94	9.08	0.27	26.94	N
Omitting Ryom et al. 2021	-1.11 [-3.38 1.17]	34	0.53	0.26	0.07	1.33	9.09	0.12	11.68	N

rstudent, Externally Standardized Residuals; dffits, DFFITS Value; cook.d, Cook's Distance; cov.r, Covariance Ratio; QE.del, Leave-One-Out τ2 and Q Values; hat, Hat Value; weight, Study Weight; Infl, Influence.

Supplementary table S5.11. Triglycerides Leave-One-Out Analysis

	**Leave-One-Out Analysis (Sorted by I2)**		**Influence Diagnostics**							
	Effect [IC 95%]	I^2	rstudent	dffits	cook.d	cov.r	QE.del	hat	weight	Infl
Omitting Seabra et al. 2016	-32.67 [-55.30; -10.04]	0	0.28	0.29	0.09	2.09	0.16	0.52	52.17	N
Omitting Vasconcellos et al. 2016	-29.46 [-45.55; -13.36]	0	-0.46	-0.11	0.01	1.06	0.03	0.06	5.48	N
Omitting Vasconcellos et al. 2020	-29.85 [-50.47; -9.24]	0	-0.07	-0.06	0.00	1.74	0.24	0.42	42.36	N

rstudent, Externally Standardized Residuals; dffits, DFFITS Value; cook.d, Cook's Distance; cov.r, Covariance Ratio; QE.del, Leave-One-Out τ2 and Q Values; hat, Hat Value; weight, Study Weight; Infl, Influence.

Supplementary table S5.12. HDL-cholesterol Leave-One-Out Analysis

	**Leave-One-Out Analysis (Sorted by I2)**		**Influence Diagnostics**							
	Effect [IC 95%]	I2	rstudent	dffits	cook.d	cov.r	QE.del	hat	weight	Infl
Omitting Seabra et al. 2016	8.83 [-1.74; 19.39]	0	-1.05	-1.31	1.71	2.56	0.60	0.61	60.93	Y
Omitting Vasconcellos et al. 2020	2.48 [-4.80; 9.76]	0	-0.06	-0.28	0.12	2.75	1.69	0.22	21.26	N
Omitting Vasconcellos et al. 2016	5.59 [-5.35; 16.53]	41	1.24	0.58	0.33	1.22	0.16	0.18	17.81	N

rstudent, Externally Standardized Residuals; dffits, DFFITS Value; cook.d, Cook's Distance; cov.r, Covariance Ratio; QE.del, Leave-One-Out τ2 and Q Values; hat, Hat Value; weight, Study Weight; Infl, Influence.

Supplementary table S5.13. Fasting Blood Glucose Leave-One-Out Analysis

	**Leave-One-Out Analysis (Sorted by I2)**		**Influence Diagnostics**							
	Effect [IC 95%]	I2	rstudent	dffits	cook.d	cov.r	QE.del	hat	weight	Infl
Omitting Seabra et al. 2016	-5.75 [-12.15; 0.65]	0	2.160	2.59	1.26	0.85	0.58	0.44	44.13	Y
Omitting Vasconcellos et al. 2016	2.01 [-2.15; 6.17]	0	-2.082	-1.63	1.13	0.36	0.91	0.31	31.18	Y
Omitting Vasconcellos et al. 2020	-1.84 [-12.17; 8.49]	80	-0.076	0.02	0.00	2.20	4.96	0.25	24.68	N

rstudent, Externally Standardized Residuals; dffits, DFFITS Value; cook.d, Cook's Distance; cov.r, Covariance Ratio; QE.del, Leave-One-Out τ2 and Q Values; hat, Hat Value; weight, Study Weight; Infl, Influence.

sResults S6. Leave-one-out analysis sssg vs other interventions.

Leave-One-Out Analysis for Physical fitness

Supplementary table S6.14. CRF leave-One-Out Analysis

	Leave-One-Out Analysis (Sorted by I2)		Influence Diagnostics							
	Effect [IC 95%]	I^2	rstudent	dffits	cook.d	cov.r	QE.del	hat	weight	Infl
Omitting Faude et al. 2010	0.021 [-0.26; 0.30]	0	0.27	0.09	0.01	1.11	0.18	0.10	10.05	N
Omitting Cvetković et al. 2018	0.053 [-0.23; 0.33]	0	-0.45	-0.15	0.02	1.11	0.05	0.10	9.57	N
Omitting Larsen et al. 2018	-0.003 [-0.60; 0.59]	0	0.13	0.26	0.07	5.10	0.24	0.80	80.38	N

rstudent, Externally Standardized Residuals; dffits, DFFITS Value; cook.d, Cook's Distance; cov.r, Covariance Ratio; QE.del, Leave-One-Out τ2 and Q Values; hat, Hat Value; weight, Study Weight; Infl, Influence.

Leave-One-Out Analysis for Anthropometric and body composition

Supplementary table S6.15. BMI leave-One-Out Analysis

	Leave-One-Out Analysis (Sorted by I^2)		Influence Diagnostics							
	Effect [IC 95%]	I^2	rstudent	dffits	cook.d	cov.r	QE.del	hat	weight	Infl
Omitting Faude et al. 2010	-0.33 [-3.32; 2.66]	0	0.10	0.08	0.01	1.72	0.02	0.42	41.87	N
Omitting Cvetković et al. 2018	-0.10 [-2.88; 2.68]	0	-0.17	-0.12	0.01	1.48	0.00	0.33	32.61	N
Omitting Seabra et al. 2016	-0.28 [-2.92; 2.36]	0	0.07	0.04	0.00	1.34	0.02	0.26	25.52	N

rstudent, Externally Standardized Residuals; dffits, DFFITS Value; cook.d, Cook's Distance; cov.r, Covariance Ratio; QE.del, Leave-One-Out τ2 and Q Values; hat, Hat Value; weight, Study Weight; Infl, Influence.

Supplementary table S6.16. Weight leave-One-Out Analysis

	Leave-One-Out Analysis (Sorted by I2)		Influence Diagnostics							
	Effect [IC 95%]	I^2	rstudent	dffits	cook.d	cov.r	QE.del	hat	weight	Infl
Omitting Faude et al. 2010	-0.608 [-3.39; 2.17]	0	0.01	0.01	0.00	1.05	0.12	0.04	4.30	N
Omitting Cvetković et al. 2018	-0.578 [-3.36; 2.20]	0	-0.05	-0.01	0.00	1.05	0.12	0.05	4.64	N
Omitting Larsen et al. 2018	-1.346 [-7.34; 4.64]	0	0.28	0.54	0.29	4.86	0.05	0.79	79.43	Y
Omitting Seabra et al. 2016	-0.423 [-3.31; 2.46]	0	-0.34	-0.12	0.02	1.13	0.01	0.12	11.62	N

rstudent, Externally Standardized Residuals; dffits, DFFITS Value; cook.d, Cook's Distance; cov.r, Covariance Ratio; QE.del, Leave-One-Out τ2 and Q Values; hat, Hat Value; weight, Study Weight; Infl, Influence.

Leave-One-Out Analysis for Cardiovascular Variables

Supplementary table S6.17. Systolic Blood Pressure Leave-One-Out Analysis

	Leave-One-Out Analysis (Sorted by I2)		Influence Diagnostics							
	Effect [IC 95%]	I^2	rstudent	dffits	cook.d	cov.r	QE.del	hat	weight	Infl
Omitting Cvetković et al. 2018	-3.88 [-7.46; -0.30]	0	1.09	0.30	0.09	1.08	0.03	0.07	6.95	N
Omitting Seabra et al. 2016	-2.78 [-7.54; 1.98]	10	-0.32	-0.48	0.40	4.28	1.11	0.73	72.50	N
Omitting Larsen et al. 2018	-2.25 [-9.39; 4.89]	10	-0.36	-0.30	0.11	1.90	1.10	0.21	20.55	N

rstudent, Externally Standardized Residuals; dffits, DFFITS Value; cook.d, Cook's Distance; cov.r, Covariance Ratio; QE.del, Leave-One-Out τ2 and Q Values; hat, Hat Value; weight, Study Weight; Infl, Influence.

Supplementary table S6.18. Diastolic Blood Pressure Leave-One-Out Analysis

	**Leave-One-Out Analysis (Sorted by I2)**		**Influence Diagnostics**							
	Effect [IC 95%]	I^2	rstudent	dffits	cook.d	cov.r	QE.del	hat	weight	Infl
Omitting Cvetković et al. 2018	0.90 [-1.20; 2.99]	0	0.05	0.01	0.00	1.04	0.00	0.03	3.44	N
Omitting Larsen et al. 2018	0.96 [-4.00; 5.91]	0	-0.02	-0.04	0.00	5.80	0.00	0.83	82.76	N
Omitting Seabra et al. 2016	0.91 [-1.31; 3.13]	0	-0.00	-0.00	0.00	1.16	0.00	0.14	13.81	N

rstudent, Externally Standardized Residuals; dffits, DFFITS Value; cook.d, Cook's Distance; cov.r, Covariance Ratio; QE.del, Leave-One-Out τ2 and Q Values; hat, Hat Value; weight, Study Weight; Infl, Influence.
